# Distinguishing Lung Adenocarcinoma from Lung Squamous Cell Carcinoma by Two Hypomethylated and Three Hypermethylated Genes: A Meta-Analysis

**DOI:** 10.1371/journal.pone.0149088

**Published:** 2016-02-10

**Authors:** Tao Huang, Jinyun Li, Cheng Zhang, Qingxiao Hong, Danjie Jiang, Meng Ye, Shiwei Duan

**Affiliations:** 1 Zhejiang Provincial Key Laboratory of Pathophysiology, School of Medicine, Ningbo University, Ningbo, Zhejiang 315211, China; 2 The Affiliated Hospital, Ningbo University, Ningbo, Zhejiang 315000, China; Duke Cancer Institute, UNITED STATES

## Abstract

Significant differences in the aberrant methylation of genes exist among various histological types of non-small cell lung cancer (NSCLC), which includes adenocarcinoma (AC) and squamous cell carcinoma (SCC). Different chemotherapeutic regimens should be administered to the two NSCLC subtypes due to their unique genetic and epigenetic profiles. The purpose of this meta-analysis was to generate a list of differentially methylated genes between AC and SCC. Our meta-analysis encompassed 151 studies on 108 genes among 12946 AC and 10243 SCC patients. Our results showed two hypomethylated genes (*CDKN2A* and *MGMT*) and three hypermethylated genes (*CDH13*, *RUNX3* and *APC*) in ACs compared with SCCs. In addition, our results showed that the pooled specificity and sensitivity values of *CDH13* and *APC* were higher than those of *CDKN2A*, *MGMT* and *RUNX3*. Our findings might provide an alternative method to distinguish between the two NSCLC subtypes.

## Introduction

Lung cancer remains the main contributor to cancer-related mortality, with 224,210 new cases and 159,260 deaths in the United States in 2014, although the incidence rate of lung cancer has been declining since the middle of 2000s [1,2]. Non-small cell lung cancer (NSCLC), accounting for almost 84% of lung cancer, includes two histological subtypes adenocarcinoma (AC) and squamous cell carcinoma (SCC), which stem from epithelial cells that line the larger airways and the peripheral small airways, respectively [2].

Differential diagnosis between AC and SCC is of clinical significance. Chemotherapy regimens for AC and SCC are different according to the guidelines of National Comprehensive Cancer Network (NCCN) for NSCLC. For instance, pemetrexed is a multiple-enzyme inhibitor, which is utilized in AC patients rather than in SCC patients [3–5]. The current methods in the differential diagnosis often involve in immunohistochemical stainings of complete surgical resection specimens. The staining proteins consist of AC positive markers (TTF-1, CK7, Muci, and Napsin A) and SCC positive markers (CK5/6, HMWCK, NTRK1/2, and p63) [6]. The sensitivity of the most widely used TTF-1 is only 62%, suggesting a need to develop new markers for the differential diagnosis [6]. Moreover, almost 25% poorly differentiated NSCLC patients cannot be classified by TTF-1, suggesting that complimentary markers are needed to enhance the specificity [7–9].

Epigenetic modifications have been shown to be an important regulatory mechanism during the multistep development of human cancers [10]. Different epigenetic modifications [11] and different microRNA and gene expression profiles were found between AC and SCC [12], suggesting that there were distinct molecular signatures between the two subtypes [13,14]. Several studies have reported that the methylation rates of *APC*, *CDH13*, *RARβ*, *LINE-1*, *RASSF1*, and *RUNX3* were significantly higher in AC than in SCC [15,16], while higher methylation frequencies of *DAPK*, *TIMP3*, *TGIF* and *SFRP4* were more often observed in SCC compared to AC [17,18]. In addition, there were significantly different chemotherapeutic outcomes between AC and SCC [19].

Due to the increasing amount of evidence, it was necessary to establish a short list of methylated genes through a comprehensive literature review. Meta-analysis can overcome the limitation of small-size samples in single study, and achieve more reliable and completed consequences through the combination and quantitative assessment of various studies [20]. In this study, we systematically reviewed the recent methylation studies and summarized the differential gene methylation between AC and SCC, and aimed to provide a handful of epigenetic clues to elaborate the molecular biomarkers of the different histological subtypes of NSCLC.

## Materials and Methods

### Identification of relevant studies

All the relevant studies, updated until January 11, 2016, were systematically searched for in the PubMed, China National Knowledge Infrastructure and Wanfang literature databases. The keywords were as follows: “(histolog* OR patholog* OR clinic*) AND lung cancer (methylation OR epigene*)”. In addition, a manual search was performed to seek other potential studies in the references of the retrieved publications.

### Inclusion and exclusion criteria

All the eligible studies should meet the following criteria: (1) the study should refer to the measurement of the gene methylation status in NSCLC patients rather than cancer cell lines; (2) the study should have sufficient methylation information on the relative genes; and (3) the study should provide detailed information on NSCLC, such as the pathological subtypes of NSCLC and the number of NSCLC subtypes. In addition, neither reviews nor abstracts were included in our analysis. Studies without detailed information on gene methylation or pathological types of NSCLC data were also excluded from the current study. The current meta-analysis was reported based on the Preferred Reporting Items for Systematic Reviews and Meta-Analysis (PRISMA) statement (S1 PRISMA Checklist).

### Data extraction

For the eligible studies, we extracted the gene, the first author’s name, the published year, the race of the study subjects, the methylation assessment method, the number of cases of AC and SCC, and the frequency of gene methylation (S1 Table).

### Statistical analysis

Review manager 5.2 software (Cochrane Collaboration, Oxford, UK) was used to calculate the combined odds ratios (ORs) and the corresponding 95% confidence intervals (95% CIs) to estimate the association in the meta-analysis. χ^2^ test was used to assess the significant heterogeneity across studies, and the result of χ^2^ test was expressed by I^2^ metric. When I^2^ metric was more than 50%, we considered that obvious heterogeneity existed in the involved studies, and a random-effect model was applied for the meta-analysis. Otherwise, a fixed-effect model was used. The aggregated sensitivity, specificity, area under the receiver operating characteristic curve (AUC) and their 95% CIs were calculated by STATA software (Stata Corporation, College Station, TX).

## Results

**Fig 1:**
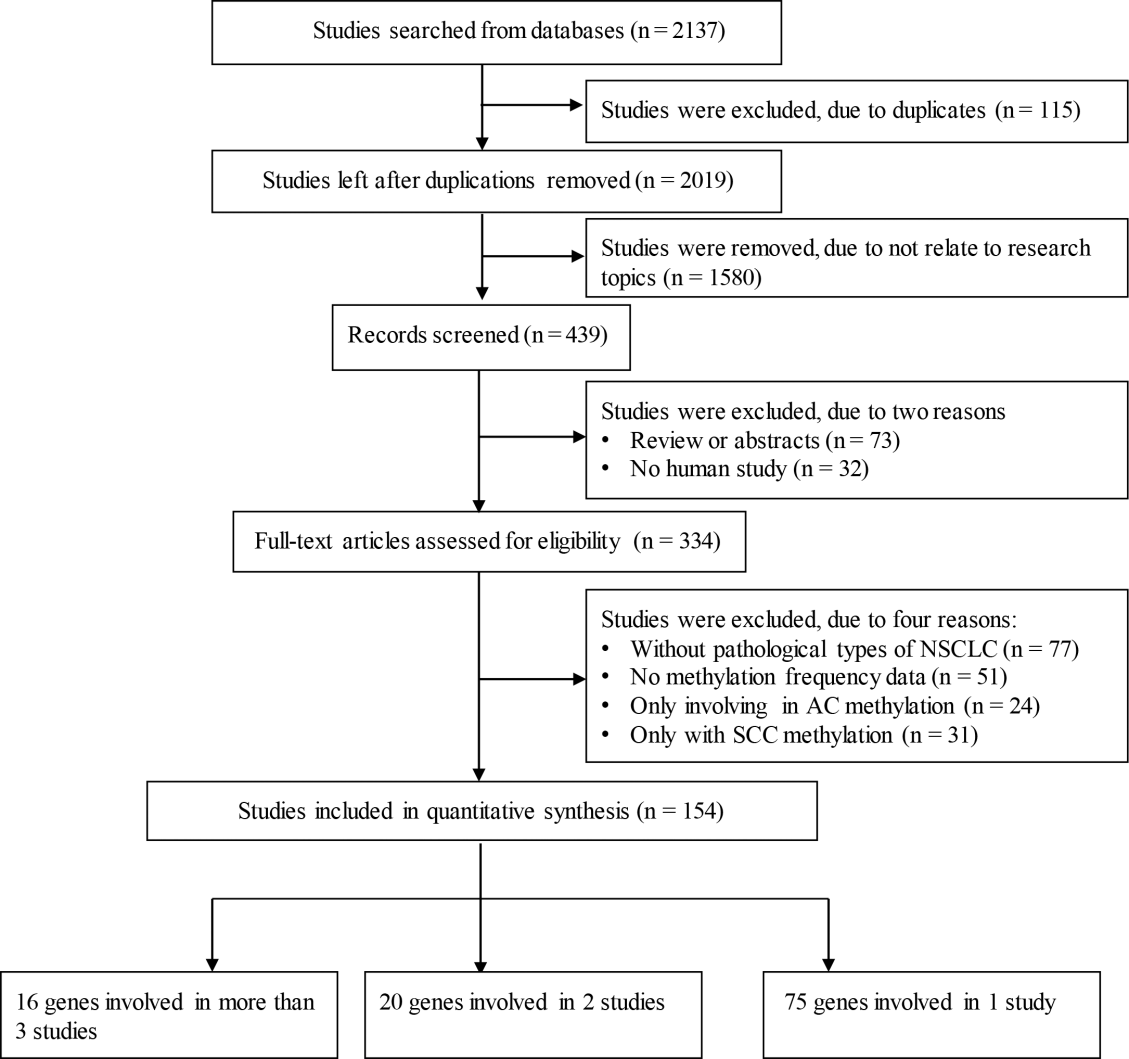
Flow diagram. The flow diagram of the stepwise selection from relevant studies.

As shown in Fig 1, a total of 2137 articles were initially retrieved from the literature databases. A filtration removed 115 duplicated publications, 1685 studies that were not human studies or full-text inaccessible studies, 77 studies without detailed information regarding pathological types of NSCLC, 51 studies without methylation frequency data, 24 studies only including AC methylation data, and 31 studies only including SCC methylation data as controls. Finally, a total of 154 eligible studies on 111 genes were included in the current meta-analysis. Among the identified genes, there were 75 genes reported by only one study, 20 genes involved in two studies, and 16 genes covered by at least three studies. The 16 genes reported by at least three studies were *CDKN2A*, *RASSF1*, *MGMT*, *MLH1*, *CDH13*, *CDH1*, *DAPK*, *RUNX3*, *APC*, *FHIT*, *SFRP1*, *RARB*, *WIF1*, *DLEC1*, *IGFBP7* and *TFPI2* (Table 1). The genes with fewer than 3 studies were listed in S2 Table.

**Table 1:** Meta-analyses of 16 gene methylation frequencies between AC and SCC.

Gene	Studies	Overall OR [95% CI]	I^2^	P Value	Median Methylation (AC/SCC, %)	25% Methylation Quartile (AC/SCC, %)	50% Methylation Quartile (AC/SCC, %)	75% Methylation Quartile (AC/SCC, %)
*CDH13*	8	2.60 [1.73, 3.90]	0%	< 0.00001	40/25	36/19	44/25	66/36
*RUNX3*	7	3.34 [2.10, 5.31]	35%	< 0.00001	36/11	27/7	36/11	41/26
*APC*	7	2.82 [1.72, 4.62]	18%	< 0.0001	62/37	43/30	63/37	73/57
*MGMT*	15	0.66 [0.52, 0.82]	0%	0.0003	32/36	29/27	32/36	40/53
*CDKN2A*	40	0.75 [0.63, 0.89]	39%	0.0008	36/49	23/33	37/49	58/57
*WIF1*	4	0.67 [0.43, 1.02]	0%	0.06	32/39	8/3	25/16	35/30
*RASSF1*	19	1.15 [0.94, 1.40]	33%	0.16	39/36	14/5	17/15	26/22
*FHIT*	6	0.82 [0.57, 1.17]	25%	0.27	27/31	7/10	14/18	23/29
*SFRP1*	5	1.23 [0.81, 1.86]	0%	0.33	37/31	9/4	11/10	36/19
*DLEC1*	4	0.80 [0.42, 1.55]	53%	0.51	34/40	8/16	12/19	25/31
*CDH1*	8	1.06 [0.63, 1.78]	22%	0.82	39/33	4/3	5/5	13/6
*DAPK*	8	1.02 [0.69, 1.51]	0%	0.92	35/36	7/6	12/9	16/12
*MLH1*	9	0.98 [0.53, 1.78]	63%	0.94	57/55	6/10	11/19	33/36
*TFPI2*	3	0.99 [0.50, 1.94]	0%	0.97	26/29	2/6	15/7	NA/NA
*RARB*	5	1.00 [0.40, 2.46]	82%	0.99	50/49	7/10	32/17	45/55
*IGFBP7*	3	1.00 [0.50, 2.00]	0%	0.99	47/47	3/1	25/4	NA/NA

NA stands for not available. From the overall OR values, *CDH13*, *RUNX3* and *APC* were significantly more methylated in AC than in SCC; *MGMT* and *CDKN2A* were significantly less methylated in AC than in SCC.

According to our systematic review, there were 5 aberrantly methylated genes (including *CDKN2A*, *MGMT*, *CDH13*, *RUNX3* and *APC*) associated with the pathological types of NSCLC, and the remaining 11 gene methylation events showed no significant difference between AC and SCC. As shown in Table 1, *CDKN2A* and *MGMT* were significantly less methylated in AC rather than in SCC, while *CDH13*, *RUNX3* and *APC* genes were significantly more methylated in AC than in SCC.

**Fig 2:**
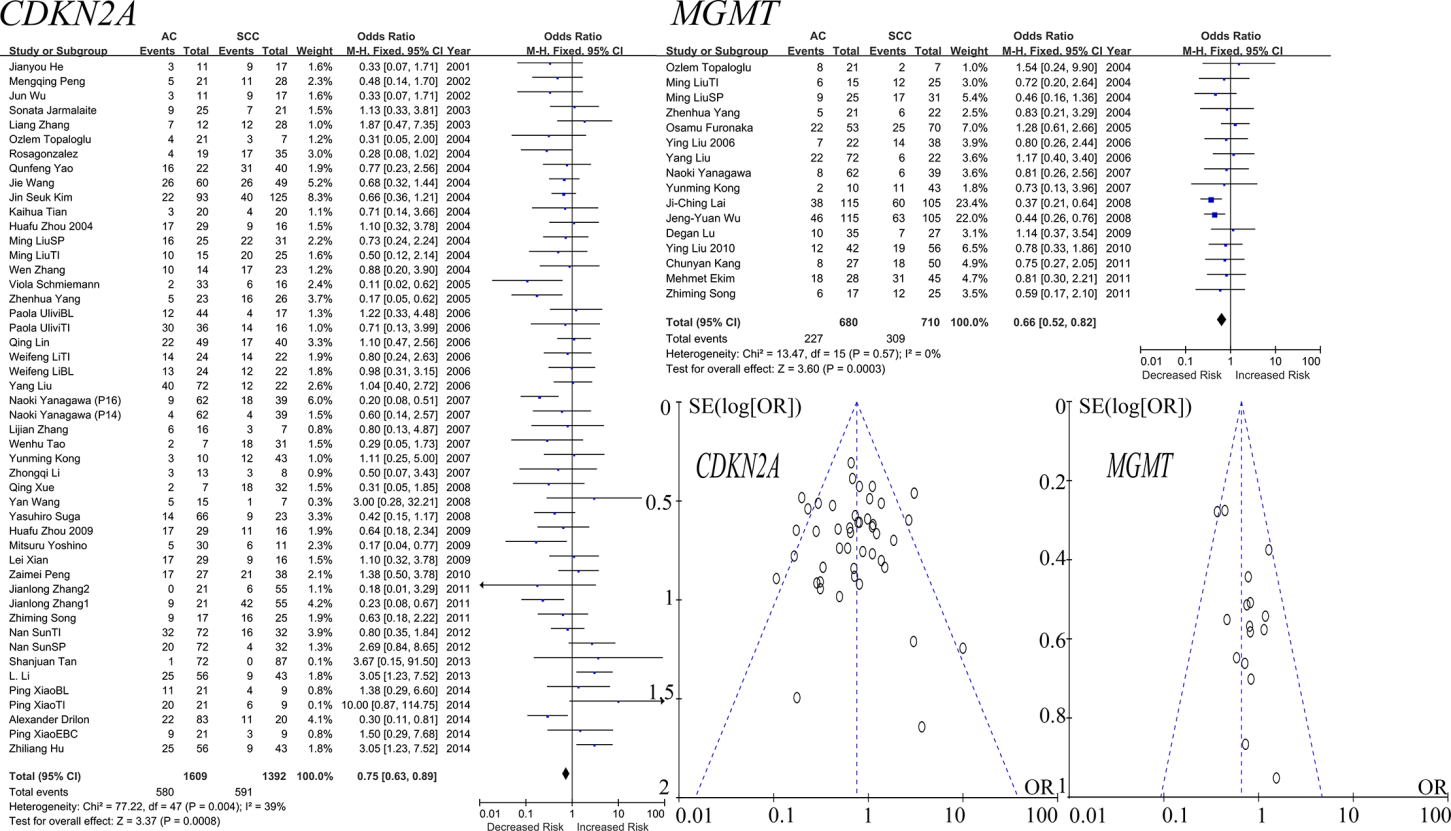
Forest and funnel plots of *CDKN2A* and *MGMT*. The forest plots of *CDKN2A* and *MGMT* displayed the effect size and 95% CIs for the included studies. Funnel plots suggested no publication bias in the meta-analyses of *CDKN2A* and *MGMT* genes. Our results showed that the total ORs for *CDKN2A* and *MGMT* were less than1, which demonstrated the methylation of *CDKN2A* and *MGMT* in AC were relatively higher than in SCC. Funnel plots of meta-analyses of *CDH13*, *RUNX3* and *APC* demonstrated no publication biases in the included studies. In addition, M-H denotes Mantel-Haenszel statistical method to calculate the combined odds ratios (ORs) and the corresponding 95% confidence intervals (95% CIs). Weight denotes the weighted average of the intervention effect estimated in each study. SE denotes standard errors.

As shown in Fig 2, the meta-analysis of *CDKN2A* methylation in 40 studies among 1609 ACs and 1392 SCCs revealed that *CDKN2A* methylation was less frequently observed in AC than in SCC (OR = 0.75, 95% CI = 0.63–0.89, P = 0.0008, I^2^ = 39%). Meta-analysis of 15 studies among 680 ACs and 710 SCCs showed that *MGMT* was significantly more methylated in SCC than in AC (OR = 0.66, 95% CI = 0.52–0.82, P = 0.0003, I^2^ = 0%).

**Fig 3:**
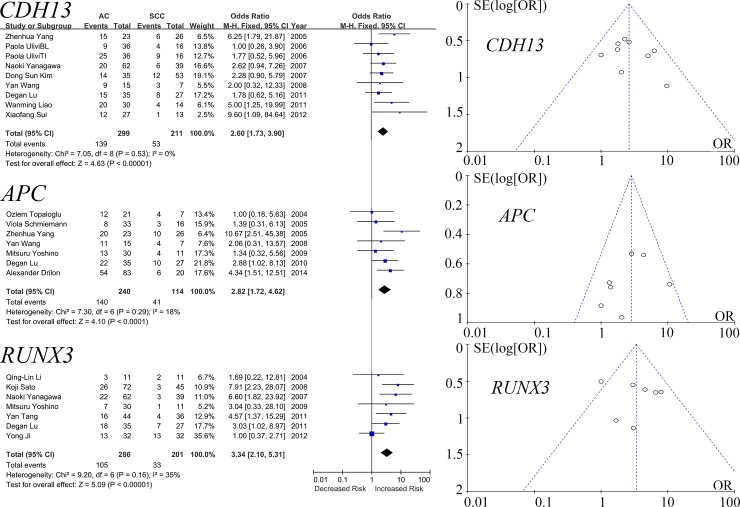
Forest and funnel plots of *CDH13*, *RUNX3* and *APC*. The forest plots of *CDH13*, *RUNX3* and *APC* displayed the effect size and 95% CIs for the included studies. Our results showed that the total ORs of *CDH13*, *RUNX3* and *APC* demonstrated that the methylation of *CDH13*, *RUNX3* and *APC* in AC were significantly more frequent than in SCC. Funnel plots of meta-analyses of *CDH13*, *RUNX3* and *APC* demonstrated no publication biases in the included studies. The details of abbreviations (M-H, ORs, CIs, and SE) and weight were shown in the legends of Fig 2.

*CDH13*, *RUNX3* and *APC* genes were shown to have significantly higher methylation frequencies in AC. Specifically, our meta-analysis of 8 studies among 299 ACs and 211 SCCs revealed that *CDH13* methylation was more frequently observed in AC than in SCC (OR = 2.60, 95% CI = 1.73–3.90, P < 0.00001, I^2^ = 0%). Meta-analysis of 7 studies among 286 ACs and 201 SCCs showed that *RUNX3* was more often methylated in SCC than in AC (OR = 3.34, 95% CI = 2.10–5.31, P < 0.00001, I^2^ = 35%). The meta-analysis of *APC* in 7 studies among 157 ACs and 94 SCCs showed that *APC* methylation was more often methylated in AC than in SCC (OR = 2.82, 95% CI = 1.72–4.62, P < 0.0001, I^2^ = 18%, Fig 3).

As shown in Table 1, the methylation of 11 genes (including *RASSF1*, *MLH1*, *CDH1*, *DAPK*, *FHIT*, *SFRP1*, *RARB*, *WIF1*, *DLEC1*, *IGFBP7* and *TFPI2*) could not distinguish between AC and SCC. And as demonstrated in Figs 2 and 3, the funnel plots of *CDKN2A*, *MGMT*, *CDH13*, *RUNX3* and *APC* indicated no significant publication bias.

Subsequently, we performed sensitivity meta-analyses of the five significant genes (Table 2). Our results showed that the pooled specificity values as differential diagnostic markers between AC and SCC for *CDH13*, *APC*, *CDKN2A*, *MGMT* and *RUNX3* were 0.74 (0.65–0.81), 0.65 (0.55–0.74), 0.55 (0.47–0.63), 0.60 (0.52–0.68) and 0.86 (0.75–0.92), respectively. The aggregated sensitivity values of *CDH13*, *APC*, *CDKN2A*, *MGMT* and *RUNX3* were 0.49 (0.38–0.59), 0.60 (0.44–0.74), 0.37 (0.29–0.45), 0.32 (0.27–0.37) and 0.47 (0.42–0.51), respectively

**Table 2:** Specificity and sensitivity of five differentially methylated genes between AC and SCC.

Gene	Specificity [95% CI]	Sensitivity [95% CI]	AUC [95% CI]
*CDH13*	0.74 [0.65, 0.81]	0.49 [0.38, 0.59]	0.68 [0.64, 0.72]
*APC*	0.65 [0.55, 0.74]	0.60 [0.44, 0.74]	0.66 [0.62, 0.70]
*CDKN2A*	0.55 [0.47, 0.63]	0.37 [0.29, 0.45]	0.45 [0.41, 0.49]
*MGMT*	0.60 [0.52, 0.68]	0.32 [0.27, 0.37]	0.40 [0.35, 0.44]
*RUNX3*	0.86 [0.75, 0.92]	0.47 [0.42, 0.51]	0.47 [0.42, 0.51]

## Discussion

Some chemotherapeutic regimens were more effective in SCC, while other drugs were more effective in non-squamous histological types [3–5]. Thus, it is necessary to differentiate the two major types of NSCLC (AC and SCC). Generally, well-differentiated AC can be identified according to the immunohistochemical staining results of TTF-1, napsin-A, and other markers [6]. However, some studies have reported that a minor fraction of poorly differentiated SCC still reacted with TTF-1 [7–9]. Our results showed that the pooled specificity and sensitivity values of *CDH13* and *APC* were higher than those of *CDKN2A*, *MGMT* and *RUNX3*. The joint effect of these methylation markers is of interest to be explored in the future.

Epigenetic modifications have been shown to account for the mechanisms in the development of different histological subtypes of cancers [21]. Besides, other studies have identified genes with significantly different methylation between different subtypes, and the differentially methylated genes (including *CDKN2A*, *APC*, *CDH13*, *THBS2* and *ERG*) have been utilized to distinguish these different histological subtypes of cancers [22,23]. Previous study has identified that *CDKN2A*, *APC* and *CDH13* have significantly different methylation frequencies between AC and SCC [23]. Another study observed that *RUNX3* methylation was significantly more often in AC than in SCC [15]. The above findings were also confirmed in the current meta-analyses. However, *MGMT* methylation frequency was not different between 77 AC and 38 SCC in the previous study [23], and this might be due to a lack of power [23]. In contrast, our meta-analyses among 680 ACs and 710 SCCs found *MGMT* methylation was significantly less in AC than in SCC.

In the current study, we identified five differentially methylated genes between AC and SCC. These five methylated genes could also be found in many other cancers. Loss of *CDH13* expression caused by promoter hypermethylation was observed in breast [24], lung [24], colorectal [25,26], prostate [27], and nasopharyngeal [28] cancers. Besides, Methylated *CDH13* could serve as a potential diagnostic and prognostic biomarker in nasopharyngeal carcinoma [28] and cervical cancer [29], respectively. Aberrantly methylated levels of *APC* and *MGMT* were also observed in colorectal cancer tissues [30]. Methylated *APC* was shown to be associated with prognostic outcomes in gastric carcinomas [31], breast cancer [32], and hepatocellular carcinoma [33]. *MGMT* was a DNA-repair gene, which greatly contributed to the microsatellite instability (MSI) in colorectal cancer [34]. Studies demonstrated that *MGMT* methylation triggered the incidence of MSI [35,36]. *CDKN2A* was a well-established gene, which played a critical role in cancer progression [37]. The inactivation of *CDKN2A* by promoter hypermethylation was observed in leukemia [38], colorectal [39], gastric [40], esophagus [41], and lung cancers [42]. Aberrantly methylated *RUNX3* was found to be associated with the risk of multiple cancers, such as hepatocellular carcinoma [43], esophageal cancer [43], gastric carcinoma [44] and NSCLC [44].

Cyclin-dependent kinase inhibitor 2A (*CDKN2A*) is known to be an important tumor suppressor gene with regulatory roles affecting CDK4 and p53 in cell cycle G1 control. This gene is frequently mutated or deleted, as well as hypermethylated, in a wide variety of tumors including NSCLC [45–47]. Interestingly, previous studies reported that the methylation status of *CDKN2A* might correlate with the response to certain chemotherapeutic drugs in breast cancer [48]. Cell line studies demonstrated that the usage of demethylating agents could reactivate *CDKN2A*, which was able to be silenced by hypermethylation [49]. Other clinical studies reported that NSCLC patients who underwent epigenetic therapy tended to have improved overall survival with statistical significance [46]. Our systematic review concluded that the methylation of *CDKN2A* was significantly more common in SCC than in AC.

*MGMT* plays a key role in regulating DNA repair via removing a methyl group from mutagenic O^6^-methylguanine, which can lead to a transition mutation through DNA replication [50]. Thus, inactivation of the O^6^-methylguanine-DNA methyltransferase (*MGMT*) gene plays an important role in the progression of cancer characterized by the accumulation of genetic changes. In addition, the epigenetic silencing of *MGMT* was shown to play a pivotal role in DNA repair pathway that was associated with cisplatin sensitivity [51]. *MGMT* promoter methylation was shown to be inversely correlated with *MGMT* expression, and silenced MGMT by promoter hypermethylation was observed in NSCLC [52]. Our meta-analysis found that the hypermethylation of *MGMT* was more common in SCC than in AC.

Cadherin 13 (*CDH13)*, also known as T-cadherin or H-cadherin (heart), is a unique member of the cadherin superfamily [53,54]. *CDH13* proteins play important roles in cell differentiation and in anti-apoptosis [55]. However, *CDH13* expression was generally down regulated by *CDH13* promoter hypermethylation in human cancers [56,57]. *CDH13* methylation was a common event in NSCLC, and it was also associated with its clinicopathological features. *CDH13* hypermethylation was observed at higher frequency in AC than in SCC [23]. Patients with *CDH13* hypermethylation tended to have lower survival [58], suggesting that *CDH13* hypermethylation could serve as a prognostic biomarker in NSCLC. The current meta-analysis also confirmed this observation.

The RUNX3 proteins belong to the runt domain-containing family of transcription factors in the regulation of gene expression [59]. Transcriptional silencing of *RUNX3* by hypermethylation was associated with various human cancers, including NSCLC [60–62]. Low *RUNX3* mRNA expression level was found to be associated with *RUNX3* promoter hypermethylation [62]. *RUNX3* hypermethylation was mostly detected in AC [53]. Further studies demonstrated that patients with higher *RUNX3* hypermethylation in AC had shorter survival even when undergoing positive treatment [63]. Our analysis indicated that *RUNX3* hypermethylation might have the potential to predict treatment outcome as a differential diagnostic marker for NSCLC subtypes.

The tumor suppressor gene adenomatous polyposis coli (*APC*) is correlated with inhibition of the Wnt signaling pathway [64]. Mutation of *APC* was shown to be associated with the emergence of colorectal cancer [65]. Decreased expression of *APC* by its promoter hypermethylation was also often observed in NSCLC [66]. Aberrant epigenetic modification of *APC* was also observed in colorectal cancer as well as in NSCLC [66,67]. The current analysis revealed that *APC* hypermethylation was more frequent in AC than in SCC.

Although our meta-analyses were performed through carefully screening numerous relevant studies, several limitations should not be underestimated. Above all, conference abstracts and inaccessible full-text articles were excluded from our meta-analyses because we were unable to retrieve relevant data for the meta-analysis. Moreover, only reports in the English or Chinese languages were chosen, and this might introduce bias in the literature selection. Meanwhile, the majority of the harvested genes with only one or two studies were excluded from this analysis. It was possible that some of them were certain specific-histology genes. Thus, future analyses of these genes in larger sample sizes were needed to confirm our findings.

In summary, our meta-analysis provided a list of differently methylated genes between AC and SCC and identified two hypomethylated (*CDKN2A* and *MGMT*) and three hypermethylated genes (*CDH13*, *RUNX3* and *APC*) that might help distinguish between AC and SCC.

## Supporting Information

S1 PRISMA Checklist The PRISMA checklist of our meta-analysis. (DOCX)

S1 Table General characteristics of all the eligible studies in the current meta-analyses. (DOC)

S2 Table Genes with less than 3 methylation studies. (DOC)
