# Supplementary material for: Distinguishing Lung Adenocarcinoma from Lung Squamous Cell Carcinoma by Two Hypomethylated and Three Hypermethylated Genes: A Meta-Analysis
Source: PLoS One. 2016 Feb 10;11(2):e0149088. doi: 10.1371/journal.pone.0149088 (PMC4749211; doi:10.1371/journal.pone.0149088)
Supplement: S1 PRISMA Checklist — (DOCX) [file pone.0149088.s001.docx]

| **Section/topic** | **#** | **Checklist item** | **Reported on page #** |
| --- | --- | --- | --- |
| **TITLE** | | |  |
| Title | 1 | Distinguishing lung adenocarcinoma from lung squamous cell carcinoma by two hypomethylated and three hypermethylated genes: a meta-analysis | Title |
| **ABSTRACT** | | |  |
| Structured summary | 2 | Background: Significant differences in the aberrant methylation of genes exist among various histological types of non-small cell lung cancer (NSCLC), which includes adenocarcinoma (AC) and squamous cell carcinoma (SCC). Different regimens should be administered to the two NSCLC subtypes due to their unique genetic and epigenetic profiles. The purpose of this meta-analysis was to generate a list of differentially methylated genes between AC and SCC.  Methods: Relevant studies were identified from PubMed, China National Knowledge Infrastructure and Wangfang literature databases. Mantel-Haenszel odds ratios and 95% confidence intervals were computed for each methylation event assuming the suitable model.  Results: Our meta-analysis encompassed 154 studies on 111 genes among 13134 AC and 10419 SCC patients. Our results showed two hypomethylated genes (*CDKN2A* and *MGMT*) and three hypermethylated genes (*CDH13*, *RUNX3* and *APC*) in ACs compared with SCCs. In addition, our results showed that the pooled specificity and sensitivity values of *CDH13* and *APC* were higher than those of *CDKN2A*, *MGMT* and *RUNX3*.  Conclusion: Our findings might provide an alternative method to distinguish between the two NSCLC subtypes. | 2 |
| **INTRODUCTION** | | |  |
| Rationale | 3 | Lung cancer remains the main contributor to cancer-related mortality, with 224,210 new cases and 159,260 deaths in the United States in 2014, although the incidence rate of lung cancer has been declining since the middle of 2000s. Non-small cell lung cancer (NSCLC), accounting for almost 84% of lung cancer, includes two histological subtypes adenocarcinoma (AC) and squamous cell carcinoma (SCC), which stem from epithelial cells that line the larger airways and the peripheral small airways, respectively. Differential diagnosis between AC and SCC is of clinical significance. Chemotherapy regimens for AC and SCC are different according to the guidelines of National Comprehensive Cancer Network (NCCN) for NSCLC. For instance, pemetrexed is a multiple-enzyme inhibitor, which is utilized in AC patients rather than in SCC patients. The current methods in the differential diagnosis often involve in immunohistochemical stainings of complete surgical resection specimens. The staining proteins consist of AC positive markers (TTF-1, CK7, Muci, and Napsin A) and SCC positive markers (CK5/6, HMWCK, NTRK1/2, and p63). The sensitivity of the most widely used TTF-1 is only 62%, suggesting a need to develop new markers for the differential diagnosis. Moreover, almost 25% poorly differentiated NSCLC patients cannot be classified by TTF-1, suggesting that complimentary markers are needed to enhance the specificity. Epigenetic modifications have been shown to be an important regulatory mechanism during the multistep development of human cancers. Different epigenetic modifications and different microRNA and gene expression profiles were found between AC and SCC, suggesting that there were distinct molecular signatures between the two subtypes. Several studies have reported that the methylation rates of *APC, CDH13, RARβ, LINE-1, RASSF1*, and *RUNX3* were significantly higher in AC than in SCC, while higher methylation frequencies of *DAPK*, *TIMP3*, *TGIF* and *SFRP4* were more often observed in SCC compared to AC. In addition, there were significantly different chemotherapeutic outcomes between AC and SCC. | 3 |
| Objectives | 4 | In this study, we systematically reviewed the recent methylation studies and summarized the different methylation profiles of genes between AC and SCC in an attempt to provide a handful of epigenetic clues to elaborate the molecular biomarkers of the different histological subtypes of NSCLC. | 3 |
| **METHODS** | | |  |
| Eligibility criteria | 5 | (1) The study should refer to the measurement of the gene methylation status in NSCLC patients rather than cancer cell lines; (2) The study should have sufficient methylation information on the relative genes; and (3) The study should provide detailed information on NSCLC, such as the pathological subtypes of NSCLC and the number of different types of NSCLC. | 4 |
| Information sources | 6 | All relevant studies, updated until January 1, 2016, were systematically searched for in the PubMed, China National Knowledge Infrastructure and Wanfang literature databases. | 4 |
| Search | 7 | The keywords were as follows: “(histolog* OR patholog* OR clinic*) AND lung cancer (methylation OR epigene*)”. In addition, a manual search was performed to seek other potential studies in the references of the retrieved publications. | 4 |
| Study selection | 8 | A total of 2137 articles were initially retrieved from the literature databases. Then, 115 duplications were eliminated, leaving 2019 studies. A further filtration removed 1685 studies that were not human studies or full-text inaccessible studies, 77 studies without detailed information regarding pathological types of NSCLC, 51 studies without methylation frequency data, 24 studies only including AC methylation data, and 31 studies only including SCC methylation data as controls. Finally, a total of 154 eligible studies on 111 genes were included in the current meta-analysis | 4 |
| Data collection process | 10 | We extracted gene, the first author’s name, the published year, race of study subjects, methylation assessment method, the number of AC and SCC, frequency of gene methylation | 4 |
| Data items | 11 | We extracted the gene, the first author’s name, the published year, the race of the study subjects, the methylation assessment method, the number of cases of AC and SCC, and the frequency of gene methylation | 4 |
| Risk of bias in individual studies | 12 | The I^2^ metric was utilized to test the heterogeneity among the included studies. When the I^2^ metric was more than 50%, we considered that obvious heterogeneity existed in the involved studies and should be adjusted by applying a random-effect model. Otherwise, a fixed-effect model was used. | 6 |
| Summary measures | 13 | Review manager 5.2 software (Cochrane Collaboration, Oxford, UK) was used to calculate the combined odds ratios (ORs) and the corresponding 95% confidence intervals (95% CIs) to estimate the association in the meta-analysis. χ2 test was used to assess the significant heterogeneity across studies, and the result of χ2 test was expressed by I2 metric. When I2 metric was more than 50%, we considered that obvious heterogeneity existed in the involved studies, and a random-effect model was applied for the meta-analysis. Otherwise, a fixed-effect model was used. The aggregated sensitivity, specificity, area under the receiver operating characteristic curve (AUC) and their 95% CIs were calculated by STATA software (Stata Corporation, College Station, TX). | 6 |
| Synthesis of results | 14 | A total of 154 eligible studies on 111 genes were included in the current meta-analysis. | 5 |

Page 1 of 2

| **Section/topic** | **#** | **Checklist item** | **Reported on page #** |
| --- | --- | --- | --- |
| Risk of bias across studies | 15 | Using Review manager 5.2, we calculated the combined odds ratios (ORs) with the corresponding 95% confidence intervals (95% CIs) to estimate the association in the meta-analysis. | 6 |
| Additional analyses | 16 | No |  |
| **RESULTS** | | |  |
| Study selection | 17 | a total of 2137 articles were initially retrieved from the literature databases. A filtration removed 115 duplicated publications, 1685 studies that were not human studies or full-text inaccessible studies, 77 studies without detailed information regarding pathological types of NSCLC, 51 studies without methylation frequency data, 24 studies only including AC methylation data, and 31 studies only including SCC methylation data as controls. Finally, a total of 154 eligible studies on 108 genes were included in the current meta-analysis. Among the identified genes, there were 75 genes reported by only one study, 20 genes involved in two studies, and 16 genes covered by at least three studies. The 16 genes reported by at least three studies were *CDKN2A, RASSF1, MGMT, MLH1, CDH13, CDH1, DAPK, RUNX3, APC, FHIT, SFRP1, RARB, WIF1, DLEC1, IGFBP7 and TFPI2* | 7 |
| Study characteristics | 18 | All eligible studies should meet the following criteria: (1) the study should refer to the measurement of the gene methylation status in NSCLC patients rather than cancer cell lines; (2) the study should have sufficient methylation information on the relative genes; and (3) the study should provide detailed information on NSCLC, such as the pathological subtypes of NSCLC and the number of different types of NSCLC. In addition, neither reviews nor abstracts were included in our analysis. Studies without detailed information on gene methylation or pathological types of NSCLC data were also omitted from our study. For the eligible studies, we extracted the gene, the first author’s name, the published year, the race of the study subjects, the methylation assessment method, the number of cases of AC and SCC, and the frequency of gene methylation (Table S1). | 4-5 |
| Risk of bias within studies | 19 | The meta-analyses showed no publication bias by funnel plot analysis. | Figure 2 and Figure 3 |
| Results of individual studies | 20 | *CDKN2A* and *MGMT* hypomethylation were correlated with AC rather than SCC (*CDKN2A*, OR = 0.75, 95% CI = 0.63-0.89, P = 0.0008, I^2^ = 39%; *MGMT*, OR = 0.66, 95% CI = 0.52-0.82, P = 0.0003, I^2^ = 0%). Hypermethylated *CDH13*, *RUNX3* and *APC* genes were associated with SCC instead of AC (*CDH13*, OR = 2.60, 95% CI = 1.73-3.90, P <0.00001, I^2^ = 0%, *RUNX3,* OR = 3.34, 95% CI = 2.10-5.31, P <0.00001, I^2^ = 35% and *APC*, OR = 2.82, 95% CI = 1.72-4.62, P < 0.0001, I^2^ = 18%). | Figure 2 and Figure 3 |
| Synthesis of results | 21 | \| Gene \| Studies \| Overall OR [95% CI] \| I^2^ \| *P* Value \| Median Methylation \| \| Methylation Quartiles \| \| \| \| \| \| \| --- \| --- \| --- \| --- \| --- \| --- \| --- \| --- \| --- \| --- \| --- \| --- \| --- \| \| 25 \| \| 50 \| \| 75 \| \| \| AC \| SCC \| AC \| SCC \| AC \| SCC \| AC \| SCC \| \| *CDH13* \| 8 \| 2.60 [1.73, 3.90] \| 0% \| < 0.00001 \| 40% \| 25% \| 36% \| 19% \| 44% \| 25% \| 66% \| 36% \| \| *RUNX3* \| 7 \| 3.34 [2.10, 5.31] \| 35% \| < 0.00001 \| 36% \| 11% \| 27% \| 7% \| 36% \| 11% \| 41% \| 26% \| \| *APC* \| 7 \| 2.82 [1.72, 4.62] \| 18% \| < 0.0001 \| 62% \| 37% \| 43% \| 30% \| 63% \| 37% \| 73% \| 57% \| \| *MGMT* \| 15 \| 0.66 [0.52, 0.82] \| 0% \| 0.0003 \| 32% \| 36% \| 29% \| 27% \| 32% \| 36% \| 40% \| 53% \| \| *CDKN2A* \| 40 \| 0.75 [0.63, 0.89] \| 39% \| 0.0008 \| 36% \| 49% \| 23% \| 33% \| 37% \| 49% \| 58% \| 57% \| \| *WIF1* \| 4 \| 0.67 [0.43, 1.02] \| 0% \| 0.06 \| 32% \| 39% \| 8% \| 3% \| 25% \| 16% \| 35% \| 30% \| \| *RASSF1* \| 19 \| 1.15 [0.94, 1.40] \| 33% \| 0.16 \| 39% \| 36% \| 14% \| 5% \| 17% \| 15% \| 26% \| 22% \| \| *FHIT* \| 6 \| 0.82 [0.57, 1.17] \| 25% \| 0.27 \| 27% \| 31% \| 7% \| 10% \| 14% \| 18% \| 23% \| 29% \| \| *SFRP1* \| 5 \| 1.23 [0.81, 1.86] \| 0% \| 0.33 \| 37% \| 31% \| 9% \| 4% \| 11% \| 10% \| 36% \| 19% \| \| *DLEC1* \| 4 \| 0.80 [0.42, 1.55] \| 53% \| 0.51 \| 34% \| 40% \| 8% \| 16% \| 12% \| 19% \| 25% \| 31% \| \| *CDH1* \| 8 \| 1.06 [0.63, 1.78] \| 22% \| 0.82 \| 39% \| 33% \| 4% \| 3% \| 5% \| 5% \| 13% \| 6% \| \| *DAPK* \| 8 \| 1.02 [0.69, 1.51] \| 0% \| 0.92 \| 35% \| 36% \| 7% \| 6% \| 12% \| 9% \| 16% \| 12% \| \| *MLH1* \| 9 \| 0.98 [0.53, 1.78] \| 63% \| 0.94 \| 57% \| 55% \| 6% \| 10% \| 11% \| 19% \| 33% \| 36% \| \| *TFPI2* \| 3 \| 0.99 [0.50, 1.94] \| 0% \| 0.97 \| 26% \| 29% \| 2% \| 6% \| 15% \| 7% \| NA \| NA \| \| *RARB* \| 5 \| 1.00 [0.40, 2.46] \| 82% \| 0.99 \| 50% \| 49% \| 7% \| 10% \| 32% \| 17% \| 45% \| 55% \| \| *IGFBP7* \| 3 \| 1.00 [0.50, 2.00] \| 0% \| 0.99 \| 47% \| 47% \| 3% \| 1% \| 25% \| 4% \| NA \| NA \| | 6-7,21 |
| Risk of bias across studies | 22 | The meta-analyses showed no publication bias by funnel plot analysis. | Figure 2 and Figure 3 |
| Additional analysis | 23 | No |  |
| **DISCUSSION** | | |  |
| Summary of evidence | 24 | In the current study, we identified five differentially methylated genes between AC and SCC. These five methylated genes could also be found in many other cancers. Previous study has identified that *CDKN2A*, *APC* and *CDH13* have significantly different methylation frequencies between AC and SCC. Another study observed that RUNX3 methylation was significantly more often in AC than in SCC. The above findings were also confirmed in the current meta-analyses. | 10 |
| Limitations | 25 | 1, Conference abstracts and inaccessible full-text articles were excluded from our meta-analyses because we were unable to retrieve relevant data for the meta-analysis.  2, Only reports in the English or Chinese languages were chosen, neglecting other languages, which introduces bias in the literature selection.  3, The majority of the harvested genes with only one or two studies were excluded from this analysis. It is possible that some of them were certain specific-histology genes | 14 |
| Conclusions | 26 | Our meta-analysis provided a list of abnormally methylated genes in AC and SCC and identified two hypomethylated (*CDKN2A* and *MGMT*) and three hypermethylated genes (*CDH13*, *RUNX3* and *APC*) that might help distinguish between AC and SCC. | 14 |
| **FUNDING** | | |  |
| Funding | 27 | The research was supported by the grants from the National Natural Science Foundation of China (31100919 and 81371469), the Natural Science Foundation of Zhejiang Province (LR13H020003), the K. C. Wong Magna Fund in Ningbo University, the Zhejiang Provincial Natural Science Foundation of China (LY16H160005), Project of Scientific Innovation Team of Ningbo (2015B11050) and the Ningbo Natural Science Foundation (2014A610235). The funders had no role in study design, data collection and analysis, decision to publish, or preparation of the manuscript. | 15 |
