# Supplementary material for: Distinguishing Lung Adenocarcinoma from Lung Squamous Cell Carcinoma by Two Hypomethylated and Three Hypermethylated Genes: A Meta-Analysis
Source: PLoS One. 2016 Feb 10;11(2):e0149088. doi: 10.1371/journal.pone.0149088 (PMC4749211; doi:10.1371/journal.pone.0149088)
Supplement: S1 Table — (DOC) [file pone.0149088.s002.doc]

S1 Table: General characteristics of all the eligible studies in the current meta-analyses

| Gene | First author / Year | Country | Ethnicity | Histology | Method | Case Sample | M+ | Total | M+ | Total |  |
| --- | --- | --- | --- | --- | --- | --- | --- | --- | --- | --- | --- |
| *CDKN2A* |  |  |  |  |  |  | AC | | SCC | |  |
|  | Jianyou He 2001 | China | Asians | NSCLC | MSP | Tissue | 3 | 11 | 9 | 17 |  |
|  | Jun Wu 2002 | China | Asians | NSCLC | MSP | Tissue | 3 | 11 | 9 | 17 |  |
|  | Mengqing Peng 2002 | China | Asians | NSCLC | PCR | Tissue | 5 | 21 | 11 | 28 |  |
|  | Liang Zhang 2003 | China | Asians | NSCLC | PCR | Tissue | 7 | 12 | 12 | 28 |  |
|  | Sonata Jarmalaite 2003 | Finland | Europeans | NSCLC | MSP | Tissue | 9 | 25 | 7 | 21 |  |
|  | Jie Wang 2004 | USA | Europeans | NSCLC | FMSP | Tissue | 26 | 60 | 26 | 49 |  |
|  | Jin Seuk Kim 2004 | Korea | Asians | NSCLC | MSP | Tissue | 22 | 93 | 40 | 125 |  |
|  | Ming Liu (TI) 2004 | China | Asians | NSCLC | MSP | Tissue | 10 | 15 | 20 | 25 |  |
|  | Ming Liu (SP) 2004 | China | Asians | NSCLC | MSP | Sputum | 16 | 25 | 22 | 31 |  |
|  | Ozlem Topaloglu 2004 | USA | Europeans | NSCLC | fRTPCR | Tissue | 4 | 21 | 3 | 7 |  |
|  | Rosagonzalez 2004 | Spain | Europeans | NSCLC | MSP | Tissue | 4 | 19 | 17 | 35 |  |
|  | Wen Zhang 2004 | China | Asians | AC+SCC | MSP | Tissue | 10 | 14 | 17 | 23 |  |
|  | Qunfeng Yao 2004 | China | Asians | NSCLC | nMsp | Blood | 16 | 22 | 31 | 40 |  |
|  | Huafu Zhou 2004 | China | Asians | NSCLC | MSP | Tissue | 17 | 29 | 9 | 16 |  |
|  | Kaihua Tian 2004 | China | Asians | NSCLC | MSP | Tissue | 3 | 20 | 4 | 20 |  |
|  | Viola Schmiemann 2005 | Germany | Europeans | NSCLC | qMSP | Tissue | 2 | 33 | 6 | 16 |  |
|  | Zhenhua Yang 2005 | China | Asians | NSCLC | MSP | Tissue | 5 | 23 | 16 | 26 |  |
|  | Paola Ulivi (BL)2006 | Italy | Europeans | NSCLC | MSP | Blood | 12 | 44 | 4 | 17 |  |
|  | Paola Ulivi (TI)2006 | Italy | Europeans | NSCLC | MSP | Tissue | 30 | 36 | 14 | 16 |  |
|  | Qing Lin 2006 | China | Asians | NSCLC | MSP | Blood | 22 | 49 | 17 | 40 |  |
|  | Yang Liu 2006 | USA | Europeans | NSCLC | MSP | Tissue | 40 | 72 | 12 | 22 |  |
|  | Weifeng Li (TI) 2006 | China | Asians | NSCLC | MSP | Tissue | 14 | 24 | 14 | 22 |  |
|  | Weifeng Li (BL) 2006 | China | Asians | NSCLC | MSP | Blood | 13 | 24 | 12 | 22 |  |
|  | Lijian Zhang 2007 | China | Asians | NSCLC | MSP | Tissue | 6 | 16 | 3 | 7 |  |
|  | Naoki Yanagawa 2007 (P16) | Japan | Asians | NSCLC | MSP | Tissue | 9 | 62 | 18 | 39 |  |
|  | Wenhu Tao 2007 | China | Asians | NSCLC | MSP | Tissue | 2 | 7 | 18 | 31 |  |
|  | Yunming Kong 2007 | China | Asians | NSCLC | nMSP | Blood | 3 | 10 | 12 | 43 |  |
|  | Zhongqi Li 2007 | China | Asians | NSCLC | MSP | Blood | 3 | 13 | 3 | 8 |  |
|  | Naoki Yanagawa 2007 (P14) | Japan | Asians | NSCLC | MSP | Tissue | 4 | 62 | 4 | 39 |  |
|  | Qing Xue 2008 | China | Asians | NSCLC | MSP | Tissue | 2 | 7 | 18 | 32 |  |
|  | Yan Wang 2008 | China | Asians | NSCLC | 3DPCR | Tissue | 5 | 15 | 1 | 7 |  |
|  | Yasuhiro Suga 2008 | Japan | Asians | NSCLC | MSP | Tissue | 14 | 66 | 9 | 23 |  |
|  | Huafu Zhou 2009 | China | Asians | NSCLC | MSP | Tissue | 17 | 29 | 11 | 16 |  |
|  | Lei Xian 2009 | China | Asians | NSCLC | MSP | Tissue | 17 | 29 | 9 | 16 |  |
|  | Mitsuru Yoshino 2009 | Japan | Asians | NSCLC | MSP | Tissue | 5 | 30 | 6 | 11 |  |
|  | Zaimei Peng 2010 | China | Asians | NSCLC | MSP | Tissue | 17 | 27 | 21 | 38 |  |
|  | Jianlong Zhang 2011 (P16) | China | Asians | NSCLC | MSP | Tissue | 9 | 21 | 42 | 55 |  |
|  | Jianlong Zhang 2011 (P14) | China | Asians | NSCLC | MSP | Tissue | 0 | 21 | 6 | 55 |  |
|  | Zhiming Song 2011 | China | Asians | NSCLC | MSP | Sputum | 9 | 17 | 16 | 25 |  |
|  | Nan Sun (TI) 2012 | China | Asians | NSCLC | MSP | Tissue | 32 | 72 | 16 | 32 |  |
|  | Nan Sun (SP) 2012 | China | Asians | NSCLC | MSP | Sputum | 20 | 72 | 4 | 32 |  |
|  | L. Li 2013 | China | Asians | NSCLC | nMSP | Tissue | 25 | 56 | 9 | 43 |  |
|  | Shanjuan Tan 2013 | China | Asians | NSCLC | MSP | Tissue | 1 | 72 | 0 | 87 |  |
|  | Zhiliang Hu 2014 | China | Asians | NSCLC | nMSP | Tissue | 25 | 56 | 9 | 43 |  |
|  | Ping Xiao (BL) 2014 | China | Asians | NSCLC | FMSP | Blood | 11 | 21 | 4 | 9 |  |
|  | Ping Xiao (EBC) 2014 | China | Asians | NSCLC | FMSP | EBC | 9 | 21 | 3 | 9 |  |
|  | Ping Xiao (TI) 2014 | China | Asians | NSCLC | FMSP | Tissue | 20 | 21 | 6 | 9 |  |
|  | Alexander Drilon2014 | USA | Europeans | NSCLC | MSP | Tissue | 22 | 83 | 11 | 20 |  |
| *RASSF1* |  |  |  |  |  |  |  |  |  |  |  |
|  | Duk-Hwan Kim 2003 | Korea | Asians | NSCLC | MSP | Tissue | 41 | 103 | 19 | 72 |  |
|  | Jie Wang 2004 | USA | Europeans | NSCLC | MSP | Tissue | 20 | 60 | 22 | 49 |  |
|  | Ozlem Topaloglu 2004 | USA | Europeans | NSCLC | fRTPCR | Tissue | 11 | 21 | 2 | 7 |  |
|  | Carmen J. Marsit 2005 | USA | Europeans | NSCLC | MSP | Tissue | 50 | 96 | 22 | 61 |  |
|  | Viola Schmiemann 2005 | Germany | Europeans | NSCLC | qMSP | Tissue | 11 | 33 | 4 | 16 |  |
|  | Hong Chen 2006 | Japan | Asians | NSCLC | MSP | Tissue | 36 | 85 | 7 | 27 |  |
|  | Naoki Yanagawa 2007 | Japan | Asians | NSCLC | MSP | Tissue | 27 | 62 | 15 | 39 |  |
|  | Zhenghong Yu 2007 | China | Asians | NSCLC | MSP | Tissue | 16 | 43 | 4 | 13 |  |
|  | Zhenhua Yang 2007 | China | Asians | NSCLC | MSP | Tissue | 15 | 25 | 15 | 28 |  |
|  | TJ Seng 2008 | Australia | Europeans | NSCLC | MSP | Tissue | 25 | 92 | 23 | 92 |  |
|  | Yan Wang 2008 | China | Asians | NSCLC | 3DPCR | Tissue | 1 | 15 | 2 | 7 |  |
|  | Zhenghong Yu 2008 | China | Asians | NSCLC | MSP | Blood | 15 | 40 | 6 | 26 |  |
|  | Degan Lu 2010 | China | Asians | NSCLC | PCR | Blood | 18 | 35 | 10 | 27 |  |
|  | Guizhi Liu 2010 | China | Asians | NSCLC | MSP | Tissue | 20 | 54 | 27 | 42 |  |
|  | Hui Zhang 2010 | China | Asians | NSCLC | MSP | Tissue | 18 | 40 | 35 | 102 |  |
|  | Zaimei Peng 2010 | China | Asians | NSCLC | MSP | Tissue | 15 | 27 | 23 | 38 |  |
|  | Haizhu Song 2011 | China | Asians | NSCLC | MSP | Tissue | 14 | 30 | 12 | 36 |  |
|  | Nan Sun (TI) 2012 | China | Asians | NSCLC | MSP | Tissue | 36 | 72 | 20 | 32 |  |
|  | Nan Sun (SP) 2012 | China | Asians | NSCLC | MSP | Sputum | 12 | 72 | 4 | 32 |  |
|  | Shanjuan Tan 2013 | China | Asians | NSCLC | MSP | Tissue | 16 | 72 | 27 | 87 |  |
|  |  |  |  |  |  |  |  |  |  |  |  |
| *MGMT* |  |  |  |  |  |  |  |  |  |  |  |
|  | Ozlem Topaloglu 2004 | USA | Europeans | NSCLC | fRTPCR | Tissue | 8 | 21 | 2 | 7 |  |
|  | Zhenhua Yang 2004 | China | Asians | NSCLC | MSP | Tissue | 5 | 21 | 6 | 22 |  |
|  | Ming Liu (TI) 2004 | China | Asians | NSCLC | MSP | Tissue | 6 | 15 | 12 | 25 |  |
|  | Ming Liu (SP) 2004 | China | Asians | NSCLC | MSP | Sputum | 9 | 25 | 17 | 31 |  |
|  | Osamu Furonaka 2005 | Japan | Asians | AC+SCC | MSP | Tissue | 22 | 53 | 25 | 70 |  |
|  | Yang Liu 2006 | USA | Europeans | NSCLC | MSP | Tissue | 22 | 72 | 6 | 22 |  |
|  | Ying Liu 2006 | China | Asians | NSCLC | MSP | Tissue | 7 | 22 | 14 | 38 |  |
|  | Yunming Kong 2007 | China | Asians | NSCLC | nMSP | Blood | 2 | 10 | 11 | 43 |  |
|  | Naoki Yanagawa 2007 | Japan | Asians | NSCLC | MSP | Tissue | 8 | 62 | 6 | 39 |  |
|  | Ji-Ching Lai 2008 | Taiwan | Asians | NSCLC | MSP | Tissue | 38 | 115 | 60 | 105 |  |
|  | Jeng-Yuan Wu 2008 | Taiwan | Asians | NSCLC | MSP | Tissue | 46 | 115 | 63 | 105 |  |
|  | Degan Lu 2009 | China | Asians | NSCLC | PCR | Blood | 10 | 35 | 7 | 27 |  |
|  | Ying Liu 2010 | China | Asians | NSCLC | MSP | Tissue | 12 | 42 | 19 | 56 |  |
|  | Zhiming Song 2011 | China | Asians | NSCLC | MSP | Sputum | 6 | 17 | 12 | 25 |  |
|  | Chunyan Kang 2011 | China | Asians | NSCLC | MSP | Tissue | 8 | 27 | 18 | 50 |  |
|  | Mehmet Ekim 2011 | Turkey | Europeans | NSCLC | MSP | Tissue | 18 | 28 | 31 | 45 |  |
|  |  |  |  |  |  |  |  |  |  |  |  |
| *MLH1* |  |  |  |  |  |  |  |  |  |  |  |
|  | Yi-Ching Wang 2003 | Taiwan | Asians | NSCLC | MSP | Tissue | 17 | 28 | 21 | 42 |  |
|  | Liang Zhang 2003 | China | Asians | NSCLC | MSP | Tissue | 8 | 12 | 19 | 28 |  |
|  | Han-Shui Hsu 2005 | Taiwan | Asians | NSCLC | MPCR | Tissue | 43 | 65 | 11 | 17 |  |
|  | Zhenhua Yang 2007 | China | Asians | NSCLC | MSP | Tissue | 11 | 23 | 16 | 26 |  |
|  | Yan Wang 2008 | China | Asians | NSCLC | 3DPCR | Tissue | 6 | 15 | 1 | 7 |  |
|  | TJ Seng 2008 | Australia | Europeans | NSCLC | MSP | Tissue | 71 | 92 | 50 | 92 |  |
|  | Xin Geng 2009 | China | Asians | NSCLC | PCR | Tissue | 23 | 32 | 61 | 84 |  |
|  | Haizhu Song 2011 | China | Asians | NSCLC | MSP | Tissue | 4 | 30 | 8 | 36 |  |
|  | A. Gomes 2014 | Portugal | Europeans | NSCLC | MSP | Tissue | 5 | 33 | 19 | 40 |  |
|  |  |  |  |  |  |  |  |  |  |  |  |
| *CDH13* |  |  |  |  |  |  |  |  |  |  |  |
|  | Zhenhua Yang 2005 | China | Asians | NSCLC | MSP | Tissue | 15 | 23 | 6 | 26 |  |
|  | Paola Ulivi (TI)2006 | Italy | Europeans | NSCLC | FMSP | Tissue | 25 | 36 | 9 | 16 |  |
|  | Paola Ulivi (BL)2006 | Italy | Europeans | NSCLC | FMSP | Blood | 9 | 36 | 4 | 16 |  |
|  | Naoki Yanagawa 2007 | Japan | Asians | NSCLC | MSP | Tissue | 20 | 62 | 6 | 39 |  |
|  | Dong Sun Kim 2007 | Korea | Asians | NSCLC | nMSP | Tissue | 14 | 35 | 12 | 53 |  |
|  | Yan Wang 2008 | China | Asians | NSCLC | 3DPCR | Tissue | 9 | 15 | 3 | 7 |  |
|  | Degan Lu 2011 | China | Asians | NSCLC | MSP | Blood | 15 | 35 | 8 | 27 |  |
|  | Wanming Liao 2011 | China | Asians | NSCLC | MSP | Tissue | 20 | 30 | 4 | 14 |  |
|  | Xiaofang Sui 2012 | China | Asians | NSCLC | MSP | Tissue | 12 | 27 | 1 | 13 |  |
|  |  |  |  |  |  |  |  |  |  |  |  |
| *CDH1* |  |  |  |  |  |  |  |  |  |  |  |
|  | Ozlem Topaloglu 2004 | USA | Europeans | NSCLC | fRTPCR | Tissue | 20 | 21 | 5 | 7 |  |
|  | Takashi Shimamoto 2004 | Japan | Asians | NSCLC | MSP | Tissue | 13 | 25 | 6 | 12 |  |
|  | Zhenhua Yang 2004 | China | Asians | NSCLC | MSP | Tissue | 4 | 21 | 6 | 22 |  |
|  | Dong Sun Kim 2007 | Korea | Asians | NSCLC | nMSP | Tissue | 11 | 35 | 19 | 53 |  |
|  | Wenwen Li 2008 | China | Asians | NSCLC | nMSP | Tissue | 5 | 7 | 3 | 16 |  |
|  | Yan Wang 2008 | China | Asians | NSCLC | 3DPCR | Tissue | 1 | 15 | 0 | 7 |  |
|  | Reng-Yun Liu 2009 | China | Asians | NSCLC | MSP | Tissue | 4 | 17 | 4 | 12 |  |
|  | Qixin Zhang 2012 | China | Asians | NSCLC | nMSP | Tissue | 4 | 17 | 5 | 15 |  |
|  |  |  |  |  |  |  |  |  |  |  |  |
| *DAPK* |  |  |  |  |  |  |  |  |  |  |  |
|  | Jun Wu 2002 | China | Asians | NSCLC | MSP | Tissue | 4 | 11 | 6 | 17 |  |
|  | Zhenhua Yang 2004 | China | Asians | NSCLC | MSP | Tissue | 7 | 21 | 8 | 22 |  |
|  | Qing Lin 2006 | China | Asians | NSCLC | MSP | Blood | 17 | 49 | 10 | 40 |  |
|  | Naoki Yanagawa 2007 | Japan | Asians | NSCLC | MSP | Tissue | 14 | 62 | 12 | 39 |  |
|  | Yan Wang 2008 | China | Asians | NSCLC | 3DPCR | Tissue | 7 | 15 | 5 | 7 |  |
|  | Zaimei Peng 2010 | China | Asians | NSCLC | MSP | Tissue | 15 | 27 | 23 | 38 |  |
|  | Degan Lu 2010 | China | Asians | NSCLC | PCR | Blood | 16 | 35 | 7 | 27 |  |
|  | Peng Chen 2012 | China | Asians | NSCLC | MSP | Blood | 9 | 32 | 9 | 29 |  |
|  |  |  |  |  |  |  |  |  |  |  |  |
| *RUNX3* |  |  |  |  |  |  |  |  |  |  |  |
|  |  |  |  |  |  |  |  |  |  |  |  |
|  | Qing-Lin Li 2004 | Korea | Asians | NSCLC | MSP | Tissue | 3 | 11 | 2 | 11 |  |
|  | Koji Sato 2006 | Japan | Asians | NSCLC | MSP | Tissue | 26 | 72 | 3 | 45 |  |
|  | Naoki Yanagawa 2007 | Japan | Asians | NSCLC | MSP | Tissue | 22 | 62 | 3 | 39 |  |
|  | Mitsuru Yoshino 2009 | Japan | Asians | NSCLC | MSP | Tissue | 7 | 30 | 1 | 11 |  |
|  | Yan Tang 2011 | China | Asians | NSCLC | nMSP | Tissue | 16 | 44 | 4 | 36 |  |
|  | Degan Lu 2011 | China | Asians | NSCLC | MSP | Blood | 18 | 35 | 7 | 27 |  |
|  | Yong Ji 2012 | China | Asians | NSCLC | MSP | Tissue | 13 | 32 | 13 | 32 |  |
|  |  |  |  |  |  |  |  |  |  |  |  |
| *APC* |  |  |  |  |  |  |  |  |  |  |  |
|  | Ozlem Topaloglu 2004 | USA | Europeans | NSCLC | fRTPCR | Tissue | 12 | 21 | 4 | 7 |  |
|  | Zhenhua Yang 2005 | China | Asians | NSCLC | MSP | Tissue | 20 | 23 | 10 | 26 |  |
|  | Viola Schmiemann 2005 | Germany | Europeans | NSCLC | qMSP | Tissue | 8 | 33 | 3 | 16 |  |
|  | Yan Wang 2008 | China | Asians | NSCLC | 3DPCR | Tissue | 11 | 15 | 4 | 7 |  |
|  | Mitsuru Yoshino 2009 | Japan | Asians | NSCLC | MSP | Tissue | 13 | 30 | 4 | 11 |  |
| *FHIT* | Degan Lu 2010  Alexander Drilon2014 | China  USA | Asians  Europeans | NSCLC  NSCLC | PCR  MSP | Blood  Tissue | 22  54 | 35  83 | 10  6 | 27  20 |  |
|  | Jin Seuk Kim 2004 | Korea | Asians | NSCLC | MSP | Tissue | 24 | 93 | 40 | 125 |  |
|  | Naoki Yanagawa 2007 | Japan | Asians | NSCLC | MSP | Tissue | 22 | 62 | 12 | 39 |  |
|  | Zhenhua Yang 2007 | China | Asians | NSCLC | MSP | Tissue | 15 | 25 | 13 | 28 |  |
|  | Hongli Li 2009 | China | Asians | NSCLC | MSP | Tissue | 9 | 22 | 22 | 30 |  |
|  | Wen Li 2010 | China | Asians | AC+SCC | MSP | Tissue | 13 | 41 | 25 | 68 |  |
|  | Shanjuan Tan 2013 | China | Asians | NSCLC | MSP | Tissue | 2 | 72 | 3 | 87 |  |
|  |  |  |  |  |  |  |  |  |  |  |  |
| *SFRP1* |  |  |  |  |  |  |  |  |  |  |  |
|  | Makoto Suzuki 2007 | Japan | Asians | NSCLC | MSP | Tissue | 44 | 135 | 28 | 87 |  |
|  | Mitsuru Yoshino 2009 | Japan | Asians | NSCLC | MSP | Tissue | 6 | 30 | 1 | 11 |  |
|  | Y. W. Zhang 2010 | China | Asians | NSCLC | MSP | Tissue | 11 | 30 | 10 | 36 |  |
|  | Haizhu Song 2010 | China | Asians | NSCLC | MSP | Tissue | 11 | 30 | 10 | 36 |  |
|  | Hanlin Fang 2012 | China | Asians | NSCLC | MSP | Tissue | 28 | 47 | 7 | 13 |  |
|  |  |  |  |  |  |  |  |  |  |  |  |
| *RARB* |  |  |  |  |  |  |  |  |  |  |  |
|  | Naoki Yanagawa 2007 | Japan | Asians | NSCLC | MSP | Tissue | 32 | 62 | 6 | 39 |  |
|  | Zhenhua Yang 2007 | China | Asians | NSCLC | MSP | Tissue | 5 | 25 | 17 | 28 |  |
|  | TJ Seng 2008 | Australia | Europeans | NSCLC | MSP | Tissue | 56 | 92 | 47 | 92 |  |
|  | Haizhu Song 2011 | China | Asians | NSCLC | MSP | Tissue | 8 | 30 | 14 | 36 |  |
|  | Cong Tan 2012 | China | Asians | NSCLC | MSP | Tissue | 34 | 58 | 63 | 104 |  |
|  |  |  |  |  |  |  |  |  |  |  |  |
| *WIF1* |  |  |  |  |  |  |  |  |  |  |  |
|  | Makoto Suzuki 2007 | Japan | Asians | NSCLC | MSP | Tissue | 30 | 135 | 28 | 87 |  |
|  | Mitsuru Yoshino 2009 | Taiwan | Asians | NSCLC | MSP | Tissue | 4 | 30 | 3 | 11 |  |
|  | Tsung-Ming Yang 2009 | Taiwan | Asians | NSCLC | MSP | Tissue | 19 | 30 | 2 | 2 |  |
|  | Su Man Lee 2013 | Korea | Asians | NSCLC | MSP | Tissue | 36 | 79 | 30 | 60 |  |
|  |  |  |  |  |  |  |  |  |  |  |  |
| *DLEC1* |  |  |  |  |  |  |  |  |  |  |  |
|  | TJ Seng 2008 | Australia | Europeans | NSCLC | MSP | Tissue | 29 | 92 | 22 | 92 |  |
|  | Youwei Zhang 2010 | China | Asians | NSCLC | MSP | Tissue | 12 | 30 | 16 | 36 |  |
|  | Yanhua Liu 2010 | China | Asians | NSCLC | MSP | Tissue | 7 | 22 | 34 | 56 |  |
|  | Haizhu Song 2011 | China | Asians | NSCLC | MSP | Tissue | 12 | 30 | 16 | 36 |  |
|  |  |  |  |  |  |  |  |  |  |  |  |
| *IGFBP7* |  |  |  |  |  |  |  |  |  |  |  |
|  | Yan Wang 2008 | China | Asians | NSCLC | 3DPCR | Tissue | 3 | 15 | 1 | 7 |  |
|  | Yuan Chen 2011 | Germany | Europeans | NSCLC | MSP | Tissue | 25 | 51 | 21 | 39 |  |
|  | Makoto Suzuki 2013 | Japan | Asians | AC+SCC | PSQ | Tissue | 26 | 47 | 4 | 9 |  |
|  |  |  |  |  |  |  |  |  |  |  |  |
| *TFPI2* |  |  |  |  |  |  |  |  |  |  |  |
|  | Duoguang Wu 2012 | China | Asians | NSCLC | MSP | Tissue | 19 | 87 | 7 | 35 |  |
|  | Ying Liang 2012 | China | Asians | NSCLC | MSP | Tissue | 2 | 10 | 7 | 17 |  |
|  | Yongqiang Dong 2013 | China | Asians | NSCLC | MSP | Tissue | 15 | 38 | 6 | 18 |  |
|  |  |  |  |  |  |  |  |  |  |  |  |
| *SFRP5* |  |  |  |  |  |  |  |  |  |  |  |
|  | Makoto Suzuki 2007 | Japan | Asians | NSCLC | MSP | Tissue | 43 | 135 | 30 | 87 |  |
|  | Mitsuru Yoshino 2009 | Japan | Asians | NSCLC | MSP | Tissue | 9 | 30 | 3 | 11 |  |
|  |  |  |  |  |  |  |  |  |  |  |  |
| *SFRP2* |  |  |  |  |  |  |  |  |  |  |  |
|  | Makoto Suzuki 2007 | Japan | Asians | NSCLC | MSP | Tissue | 80 | 135 | 32 | 87 |  |
|  | Mitsuru Yoshino 2009 | Japan | Asians | NSCLC | MSP | Tissue | 20 | 30 | 3 | 11 |  |
|  |  |  |  |  |  |  |  |  |  |  |  |
| *LINE-1* |  |  |  |  |  |  |  |  |  |  |  |
|  | Kenichiro Saito 2010 | Japan | Asians | NSCLC | RTMSP | Tissue | 134 | 152 | 56 | 87 |  |
|  | Makoto Suzuki 2013 | Japan | Asians | AC+SCC | PSQ | Tissue | 25 | 47 | 6 | 9 |  |
|  |  |  |  |  |  |  |  |  |  |  |  |
| *IL-12RB2* |  |  |  |  |  |  |  |  |  |  |  |
|  | Makoti Suzuki 2007 | Japan | Asians | NSCLC | MSP | Tissue | 48 | 133 | 41 | 82 |  |
|  | Mitsuru Yoshino 2009 | Japan | Asians | NSCLC | MSP | Tissue | 13 | 30 | 7 | 11 |  |
|  |  |  |  |  |  |  |  |  |  |  |  |
| *TIMP3* |  |  |  |  |  |  |  |  |  |  |  |
|  | Nokia Yatagan 2007 | Japan | Asians | NSCLC | MSP | Tissue | 11 | 62 | 1 | 39 |  |
|  | Yan Wang 2008 | China | Asians | NSCLC | 3DPCR | Tissue | 6 | 15 | 3 | 7 |  |
|  |  |  |  |  |  |  |  |  |  |  |  |
| *IGSF4* |  |  |  |  |  |  |  |  |  |  |  |
|  | G Heller 2006 | Australia | Europeans | NSCLC | MSP | Tissue | 42 | 133 | 50 | 109 |  |
|  | van den Berg 2011 (M1) | Netherlands | Europeans | NSCLC | MSP | Tissue | 9 | 11 | 11 | 19 |  |
|  | van den Berg 2011 (M5) | Netherlands | Europeans | NSCLC | MSP | Tissue | 4 | 11 | 10 | 19 |  |
|  | van den Berg 2011 (M9) | Netherlands | Europeans | NSCLC | MSP | Tissue | 10 | 11 | 14 | 19 |  |
|  |  |  |  |  |  |  |  |  |  |  |  |
| *RARB2* |  |  |  |  |  |  |  |  |  |  |  |
|  | Ozlem Topaloglu 2004 | USA | Europeans | NSCLC | fRTPCR | Tissue | 2 | 21 | 1 | 7 |  |
|  | Viola Schmiemann 2005 | Germany | Europeans | NSCLC | qMSP | Tissue | 11 | 33 | 8 | 15 |  |
|  |  |  |  |  |  |  |  |  |  |  |  |
| *PTGER2* |  |  |  |  |  |  |  |  |  |  |  |
|  | Lei Tian 2008 | Japan | Asians | NSCLC | MSP | Tissue | 86 | 133 | 41 | 85 |  |
|  | Mitsuru Yoshino 2009 | Japan | Asians | NSCLC | MSP | Tissue | 22 | 30 | 7 | 11 |  |
|  |  |  |  |  |  |  |  |  |  |  |  |
| *BRCA1* |  |  |  |  |  |  |  |  |  |  |  |
|  | Carmen J Marist 2004 | USA | Europeans | NSCLC | MSP | Tissue | 5 | 84 | 0 | 60 |  |
|  | Yan Wang 2008 | China | Asians | NSCLC | 3DPCR | Tissue | 2 | 15 | 1 | 7 |  |
|  |  |  |  |  |  |  |  |  |  |  |  |
| *MSH2* |  |  |  |  |  |  |  |  |  |  |  |
|  | Han-Shui Hsu 2005 | Taiwan | Asians | NSCLC | MPCR | Tissue | 25 | 65 | 6 | 17 |  |
|  | A. Gomes 2014 | Portugal | Europeans | NSCLC | MSP | Tissue | 14 | 33 | 12 | 40 |  |
|  |  |  |  |  |  |  |  |  |  |  |  |
| *CXCL12* |  |  |  |  |  |  |  |  |  |  |  |
|  | Makoto Suzuki 2008 | Japan | Asians | NSCLC | MSP | Tissue | 46 | 134 | 33 | 87 |  |
|  | Mitsuru Yoshino 2009 | Japan | Asians | NSCLC | MSP | Tissue | 10 | 30 | 6 | 11 |  |
|  |  |  |  |  |  |  |  |  |  |  |  |
| *CHFR* |  |  |  |  |  |  |  |  |  |  |  |
|  | Masafumi Takeshita 2010 | Japan | Asians | NSCLC | MSP | Tissue | 2 | 48 | 9 | 20 |  |
|  | Takaomi Koga 2011 | Japan | Asians | NSCLC | MSP | Tissue | 16 | 165 | 12 | 40 |  |
|  |  |  |  |  |  |  |  |  |  |  |  |
| *BLU* |  |  |  |  |  |  |  |  |  |  |  |
|  | Carmen J. Marsit 2005 | USA | Europeans | NSCLC | MSP | Tissue | 43 | 83 | 20 | 57 |  |
|  | TJ Seng 2008 | Australia | Europeans | NSCLC | MSP | Tissue | 42 | 92 | 25 | 92 |  |
|  |  |  |  |  |  |  |  |  |  |  |  |
| *EPB41L3* |  |  |  |  |  |  |  |  |  |  |  |
|  | G Heller 2006 | Australia | Europeans | NSCLC | MSP | Tissue | 75 | 133 | 60 | 109 |  |
|  | Shinji Kikuchi 2005 | Japan | Asians | AC+SCC | bisulfite SSCP | Tissue | 34 | 68 | 19 | 26 |  |
|  |  |  |  |  |  |  |  |  |  |  |  |
| *BRMS1* |  |  |  |  |  |  |  |  |  |  |  |
|  | Jiyun Yang 2011 | China | Asians | NSCLC | MSP | Tissue | 64 | 155 | 81 | 150 |  |
|  | I Balgkouranidou (TI) 2014 | Greece | Europeans | NSCLC | MSP | Tissue | 15 | 23 | 14 | 25 |  |
|  | I Balgkouranidou (BL) 2014 | Greece | Europeans | NSCLC | RTMSP | Blood | 13 | 20 | 11 | 23 |  |
|  |  |  |  |  |  |  |  |  |  |  |  |
| *SEPT9* |  |  |  |  |  |  |  |  |  |  |  |
|  | Rejane Hughes Carvalho 2013 | Netherlands | Europeans | NSCLC | MSP | Tissue | 4 | 18 | 2 | 27 |  |
|  | Tomasz Powro´zek 2014 | Poland | Europeans | NSCLC | MSP | Tissue | 11 | 20 | 12 | 20 |  |
|  |  |  |  |  |  |  |  |  |  |  |  |
| *PTEN* |  |  |  |  |  |  |  |  |  |  |  |
|  | Dong Sun Kim 2010 | Korea | Asians | NSCLC | MSP | Tissue | 7 | 78 | 4 | 59 |  |
|  | Zhenxue Bao 2013 | China | Asians | NSCLC | MSP | Tissue | 25 | 47 | 23 | 36 |  |
|  |  |  |  |  |  |  |  |  |  |  |  |
| *Dkk3* |  |  |  |  |  |  |  |  |  |  |  |
|  | Makoti Suzuki 2007 | Japan | Asians | NSCLC | MSP | Tissue | 22 | 135 | 7 | 87 |  |
|  | Yanrong Lei 2013 | China | Asians | NSCLC | MSP | Blood | 15 | 28 | 19 | 35 |  |
|  |  |  |  |  |  |  |  |  |  |  |  |
| *HIN-1* |  |  |  |  |  |  |  |  |  |  |  |
|  | Hisayuki Shigematsu 2005 | USA | Asians | NSCLC | MSP | Tissue | 51 | 199 | 42 | 132 |  |
|  | Chunhui Zhou 2012 | China | Asians | NSCLC | MSP | Tissue | 23 | 32 | 24 | 35 |  |
|  |  |  |  |  |  |  |  |  |  |  |  |
| *TMS1* |  |  |  |  |  |  |  |  |  |  |  |
|  | Conghui Wang2008 | China | Asians | NSCLC | MSP | Tissue | 8 | 19 | 9 | 24 |  |
|  | Degan Lu 2013 | China | Asians | NSCLC | MSP | Blood | 10 | 35 | 4 | 27 |  |
|  |  |  |  |  |  |  |  |  |  |  |  |
| *GSTP1* |  |  |  |  |  |  |  |  |  |  |  |
|  | Ozlem Topaloglu 2004 | USA | Europeans | NSCLC | fRTPCR | Tissue | 3 | 21 | 0 | 7 |  |
|  |  |  |  |  |  |  |  |  |  |  |  |
| *IGFBP3* |  |  |  |  |  |  |  |  |  |  |  |
|  | Yoon Soo Chang 2002 | USA | Europeans | NSCLC | MSP | Tissue | 28 | 40 | 21 | 36 |  |
|  |  |  |  |  |  |  |  |  |  |  |  |
| *CDKN2B* |  |  |  |  |  |  |  |  |  |  |  |
|  | Yan Wang 2008 | China | Asians | NSCLC | 3DPCR | Tissue | 1 | 15 | 0 | 7 |  |
|  |  |  |  |  |  |  |  |  |  |  |  |
| *HPP1* |  |  |  |  |  |  |  |  |  |  |  |
|  | Mitsuru Yoshino 2009 | Japan | Asians | NSCLC | MSP | Tissue | 8 | 30 | 3 | 11 |  |
|  |  |  |  |  |  |  |  |  |  |  |  |
| *DRM/Gremlin* |  |  |  |  |  |  |  |  |  |  |  |
|  | Mitsuru Yoshino 2009 | Japan | Asians | NSCLC | MSP | Tissue | 19 | 30 | 9 | 11 |  |
|  |  |  |  |  |  |  |  |  |  |  |  |
| *Reprimo* |  |  |  |  |  |  |  |  |  |  |  |
|  | Mitsuru Yoshino 2009 | Japan | Asians | NSCLC | MSP | Tissue | 10 | 30 | 7 | 11 |  |
|  |  |  |  |  |  |  |  |  |  |  |  |
| *SPARC* |  |  |  |  |  |  |  |  |  |  |  |
|  | Mitsuru Yoshino 2009 | Japan | Asians | NSCLC | MSP | Tissue | 8 | 30 | 3 | 11 |  |
|  |  |  |  |  |  |  |  |  |  |  |  |
| *EDNRB* |  |  |  |  |  |  |  |  |  |  |  |
|  | Shu-Chen Chen 2006 | Taiwan | Asians | AC+SCC | MSP | Tissue | 7 | 34 | 19 | 45 |  |
|  |  |  |  |  |  |  |  |  |  |  |  |
| *KEAP1* |  |  |  |  |  |  |  |  |  |  |  |
|  | Lucia Anna Muscarella 2011 | Italy | Europeans | NSCLC | qMSP | Tissue | 8 | 11 | 13 | 15 |  |
|  |  |  |  |  |  |  |  |  |  |  |  |
| *EFEMP1* |  |  |  |  |  |  |  |  |  |  |  |
|  | Rui Wang 2010 | China | Asians | NSCLC | MSP | Tissue | 13 | 28 | 7 | 19 |  |
|  |  |  |  |  |  |  |  |  |  |  |  |
| *SLIT2* |  |  |  |  |  |  |  |  |  |  |  |
|  | Makoto Suzuki 2013 | Japan | Asians | AC+SCC | PSQ | Tissue | 32 | 47 | 4 | 9 |  |
|  |  |  |  |  |  |  |  |  |  |  |  |
| *CYGB* |  |  |  |  |  |  |  |  |  |  |  |
|  | George Xinarianos 2006 | UK | Europeans | NSCLC | PSQ | Tissue | 15 | 25 | 9 | 27 |  |
|  |  |  |  |  |  |  |  |  |  |  |  |
| *ESR1* |  |  |  |  |  |  |  |  |  |  |  |
|  | Yasuhiro Suga 2008 | Japan | Asians | NSCLC | MSP | Tissue | 35 | 66 | 11 | 23 |  |
|  |  |  |  |  |  |  |  |  |  |  |  |
| *SEMA3B* |  |  |  |  |  |  |  |  |  |  |  |
|  | Tamotsu Kuroki 2003 | USA | Europeans | NSCLC | MSP | Tissue | 5 | 10 | 4 | 11 |  |
|  |  |  |  |  |  |  |  |  |  |  |  |
| *MIR9-3* |  |  |  |  |  |  |  |  |  |  |  |
|  | Gerwin Heller 2012 | Australia | Europeans | NSCLC | MS-HRM | Tissue | 46 | 57 | 19 | 40 |  |
|  |  |  |  |  |  |  |  |  |  |  |  |
| *RRAD* |  |  |  |  |  |  |  |  |  |  |  |
|  | Makoto Suzuki 2007 | Japan | Mix | NSCLC | MSP | Tissue | 43 | 115 | 37 | 75 |  |
|  |  |  |  |  |  |  |  |  |  |  |  |
| *RASGRF2* |  |  |  |  |  |  |  |  |  |  |  |
|  | Hong Chen 2006 | Japan | Asians | NSCLC | MSP | Tissue | 28 | 85 | 11 | 27 |  |
|  |  |  |  |  |  |  |  |  |  |  |  |
| *TNFRSF10C* |  |  |  |  |  |  |  |  |  |  |  |
|  | Narayan Shivapurkar 2004 | USA | Europeans | AC+SCC | MSP | Tissue | 3 | 26 | 1 | 14 |  |
|  |  |  |  |  |  |  |  |  |  |  |  |
| *FANCF* |  |  |  |  |  |  |  |  |  |  |  |
|  | Carmen J Marsit 2004 | USA | Europeans | NSCLC | MSP | Tissue | 8 | 69 | 10 | 57 |  |
|  |  |  |  |  |  |  |  |  |  |  |  |
| *DAB2IP* |  |  |  |  |  |  |  |  |  |  |  |
|  | Masaaki Yano 2005 (m2a) | Japan | Asians | NSCLC | MSP | Tissue | 15 | 48 | 10 | 21 |  |
|  | Masaaki Yano 2005 (m2b) | Japan | Asians | NSCLC | MSP | Tissue | 15 | 48 | 9 | 21 |  |
|  | Masaaki Yano 2005 (m2a+2b) | Japan | Asians | NSCLC | MSP | Tissue | 13 | 48 | 9 | 21 |  |
|  |  |  |  |  |  |  |  |  |  |  |  |
| *RXRG* |  |  |  |  |  |  |  |  |  |  |  |
|  | Su Man Lee 2010 | Korea | Asians | NSCLC | MSP | Tissue | 18 | 79 | 15 | 60 |  |
|  |  |  |  |  |  |  |  |  |  |  |  |
| *RGC32* |  |  |  |  |  |  |  |  |  |  |  |
|  | Dong Sun Kim 2011 | Korea | Asians | NSCLC | MSP | Tissue | 34 | 117 | 11 | 56 |  |
|  |  |  |  |  |  |  |  |  |  |  |  |
| *GADD45A* |  |  |  |  |  |  |  |  |  |  |  |
|  | Yeon Kyung Na 2010 | China | Asians | NSCLC | MSP | Tissue | 2 | 79 | 0 | 60 |  |
|  |  |  |  |  |  |  |  |  |  |  |  |
| *GADD45B* |  |  |  |  |  |  |  |  |  |  |  |
|  | Yeon Kyung Na 2010 | China | Asians | NSCLC | MSP | Tissue | 3 | 79 | 7 | 60 |  |
|  |  |  |  |  |  |  |  |  |  |  |  |
| *GADD45G* |  |  |  |  |  |  |  |  |  |  |  |
|  | Yeon Kyung Na 2010 | China | Asians | NSCLC | MSP | Tissue | 25 | 79 | 19 | 60 |  |
|  |  |  |  |  |  |  |  |  |  |  |  |
| *HOXA5* |  |  |  |  |  |  |  |  |  |  |  |
|  | Dong-Sun Kim 2009 | Korea | Asians | NSCLC | MSP | Tissue | 66 | 79 | 47 | 60 |  |
|  |  |  |  |  |  |  |  |  |  |  |  |
| *NGB* |  |  |  |  |  |  |  |  |  |  |  |
|  | Urszula Oleksiewicz 2011 | UK | Europeans | NSCLC | PSQ | Tissue | 36 | 69 | 16 | 90 |  |
|  |  |  |  |  |  |  |  |  |  |  |  |
| *sFRP* |  |  |  |  |  |  |  |  |  |  |  |
|  | Makoto Suzuki 2007 | Japan | Asians | NSCLC | MSP | Tissue | 96 | 135 | 53 | 87 |  |
|  |  |  |  |  |  |  |  |  |  |  |  |
| *RXRA* |  |  |  |  |  |  |  |  |  |  |  |
|  | Su Man Lee 2010 | Korea | Asians | NSCLC | MSP | Tissue | 5 | 79 | 3 | 60 |  |
|  |  |  |  |  |  |  |  |  |  |  |  |
| *RXRB* |  |  |  |  |  |  |  |  |  |  |  |
|  | Su Man Lee 2010 | Korea | Asians | NSCLC | MSP | Tissue | 2 | 79 | 4 | 60 |  |
|  |  |  |  |  |  |  |  |  |  |  |  |
| *MIR-193a* |  |  |  |  |  |  |  |  |  |  |  |
|  | Gerwin Heller 2012 | Australia | Europeans | NSCLC | MS-HRM | Tissue | 21 | 57 | 20 | 40 |  |
|  |  |  |  |  |  |  |  |  |  |  |  |
| *Axin* |  |  |  |  |  |  |  |  |  |  |  |
|  | Lian-He Yang 2013 | China | Asians | AC+SCC | nMSP | Tissue | 14 | 36 | 15 | 31 |  |
|  |  |  |  |  |  |  |  |  |  |  |  |
| *MAL* |  |  |  |  |  |  |  |  |  |  |  |
|  | Makoto Suzuki 2013 | Japan | Asians | AC+SCC | PSQ | Tissue | 21 | 47 | 5 | 9 |  |
|  |  |  |  |  |  |  |  |  |  |  |  |
| *LKB1* |  |  |  |  |  |  |  |  |  |  |  |
|  | Su Man Lee 2013 | Korea | Asians | NSCLC | nMSP | Tissue | 15 | 105 | 6 | 54 |  |
|  |  |  |  |  |  |  |  |  |  |  |  |
| *MIR-503* |  |  |  |  |  |  |  |  |  |  |  |
|  | Ning Li 2014 | China | Asians | AC+SCC | COBRA | Tissue | 24 | 31 | 28 | 34 |  |
|  |  |  |  |  |  |  |  |  |  |  |  |
| *CD44* |  |  |  |  |  |  |  |  |  |  |  |
|  | Yan Wang 2008 | China | Asians | NSCLC | MSP | Tissue | 2 | 15 | 1 | 7 |  |
|  |  |  |  |  |  |  |  |  |  |  |  |
| *TERT* |  |  |  |  |  |  |  |  |  |  |  |
|  | Yan Wang 2008 | China | Asians | NSCLC | 3DPCR | Tissue | 5 | 15 | 3 | 7 |  |
|  |  |  |  |  |  |  |  |  |  |  |  |
| *CALCA* |  |  |  |  |  |  |  |  |  |  |  |
|  | Yan Wang 2008 | China | Asians | NSCLC | 3DPCR | Tissue | 11 | 15 | 6 | 7 |  |
|  |  |  |  |  |  |  |  |  |  |  |  |
| *ER* |  |  |  |  |  |  |  |  |  |  |  |
|  | Yan Wang 2008 | China | Asians | NSCLC | 3DPCR | Tissue | 8 | 15 | 2 | 7 |  |
|  |  |  |  |  |  |  |  |  |  |  |  |
| *TNFRSF10D* |  |  |  |  |  |  |  |  |  |  |  |
|  | Narayan Shivapurkar 2004 | USA | Europeans | AC+SCC | MSP | Tissue | 4 | 26 | 3 | 14 |  |
|  |  |  |  |  |  |  |  |  |  |  |  |
| *ASPP1* |  |  |  |  |  |  |  |  |  |  |  |
|  | Wanli Wei 2011 | China | Asians | NSCLC | MSP | Tissue | 22 | 47 | 13 | 33 |  |
|  |  |  |  |  |  |  |  |  |  |  |  |
| *p57KIP2* |  |  |  |  |  |  |  |  |  |  |  |
|  | Takaya Kobatake 2004 | Japan | Asians | NSCLC | MSP | Tissue | 18 | 55 | 7 | 22 |  |
|  |  |  |  |  |  |  |  |  |  |  |  |
| *PRSS3* |  |  |  |  |  |  |  |  |  |  |  |
|  | Carmen J. Marsit 2005 | USA | Europeans | NSCLC | MSP | Tissue | 47 | 87 | 33 | 58 |  |
|  |  |  |  |  |  |  |  |  |  |  |  |
| *XPC* |  |  |  |  |  |  |  |  |  |  |  |
|  | Y-H Wu 2007 | Taiwan | Asians | AC+SCC | MSP | Tissue | 54 | 82 | 51 | 76 |  |
|  |  |  |  |  |  |  |  |  |  |  |  |
| *RASSF2* |  |  |  |  |  |  |  |  |  |  |  |
|  | Kyoichi Kaira 2007 | Japan | Asians | NSCLC | MSP | Tissue | 19 | 57 | 11 | 42 |  |
|  |  |  |  |  |  |  |  |  |  |  |  |
| *HS3ST2* |  |  |  |  |  |  |  |  |  |  |  |
|  | Jung-Ah Hwang 2013 | korea | Asians | NSCLC | MS-HRM | Tissue | 48 | 125 | 39 | 146 |  |
|  |  |  |  |  |  |  |  |  |  |  |  |
| *ZIC4* |  |  |  |  |  |  |  |  |  |  |  |
|  | Rejane Hughes Carvalho 2013 | Netherlands | Europeans | NSCLC | MSP | Tissue | 9 | 16 | 23 | 27 |  |
|  |  |  |  |  |  |  |  |  |  |  |  |
| *DOT1L* |  |  |  |  |  |  |  |  |  |  |  |
|  | Rejane Hughes Carvalho 2013 | Netherlands | Europeans | NSCLC | MSP | Tissue | 15 | 19 | 27 | 27 |  |
|  |  |  |  |  |  |  |  |  |  |  |  |
| *LIMK1* |  |  |  |  |  |  |  |  |  |  |  |
|  | Rejane Hughes Carvalho 2013 | Netherlands | Europeans | NSCLC | MSP | Tissue | 6 | 18 | 17 | 25 |  |
|  |  |  |  |  |  |  |  |  |  |  |  |
| *EN1* |  |  |  |  |  |  |  |  |  |  |  |
|  | Carvalho 2013 (Frag_01) | Netherlands | Europeans | NSCLC | MSP | Tissue | 7 | 14 | 21 | 26 |  |
|  | Carvalho 2013 (Frag_02) | Netherlands | Europeans | NSCLC | MSP | Tissue | 11 | 18 | 20 | 24 |  |
|  |  |  |  |  |  |  |  |  |  |  |  |
| *MSC* |  |  |  |  |  |  |  |  |  |  |  |
|  | Rejane Hughes Carvalho 2013 | Netherlands | Europeans | NSCLC | MSP | Tissue | 11 | 18 | 4 | 27 |  |
|  |  |  |  |  |  |  |  |  |  |  |  |
| *GAS1* |  |  |  |  |  |  |  |  |  |  |  |
|  | Rejane Hughes Carvalho 2013 | Netherlands | Europeans | NSCLC | MSP | Tissue | 18 | 20 | 14 | 25 |  |
|  |  |  |  |  |  |  |  |  |  |  |  |
| *HOXA1* |  |  |  |  |  |  |  |  |  |  |  |
|  | Rejane Hughes Carvalho 2013 | Netherlands | Europeans | NSCLC | MSP | Tissue | 8 | 19 | 3 | 25 |  |
|  |  |  |  |  |  |  |  |  |  |  |  |
| *FAM78B* |  |  |  |  |  |  |  |  |  |  |  |
|  | Rejane Hughes Carvalho 2013 | Netherlands | Europeans | NSCLC | MSP | Tissue | 14 | 20 | 5 | 27 |  |
|  |  |  |  |  |  |  |  |  |  |  |  |
| *MYOD1* |  |  |  |  |  |  |  |  |  |  |  |
|  | Zhenhua Yang 2004 | China | Asians | NSCLC | MSP | Tissue | 10 | 21 | 7 | 22 |  |
|  |  |  |  |  |  |  |  |  |  |  |  |
| *WT1* |  |  |  |  |  |  |  |  |  |  |  |
|  | Zhenhua Yang 2004 | China | Asians | NSCLC | MSP | Tissue | 10 | 21 | 12 | 22 |  |
|  |  |  |  |  |  |  |  |  |  |  |  |
| *TP73* |  |  |  |  |  |  |  |  |  |  |  |
|  | Zhenhua Yang 2005 | China | Asians | NSCLC | MSP | Tissue | 13 | 23 | 9 | 26 |  |
|  |  |  |  |  |  |  |  |  |  |  |  |
| *AR* |  |  |  |  |  |  |  |  |  |  |  |
|  | Zhenhua Yang 2004 | China | Asians | NSCLC | MSP | Tissue | 7 | 21 | 3 | 22 |  |
|  |  |  |  |  |  |  |  |  |  |  |  |
| *p21WAF* |  |  |  |  |  |  |  |  |  |  |  |
|  | Zhenhua Yang 2004 | China | Asians | NSCLC | MSP | Tissue | 4 | 21 | 8 | 22 |  |
|  |  |  |  |  |  |  |  |  |  |  |  |
| *0CT6* |  |  |  |  |  |  |  |  |  |  |  |
|  | Zhenhua Yang 2004 | China | Asians | NSCLC | MSP | Tissue | 5 | 21 | 5 | 22 |  |
|  |  |  |  |  |  |  |  |  |  |  |  |
| *EPHB4* |  |  |  |  |  |  |  |  |  |  |  |
|  | Xiaocheng Wang (TI) 2009 | China | Asians | NSCLC | MSP | Tissue | 10 | 28 | 9 | 32 |  |
|  | Xiaocheng Wang (BL) 2009 | China | Asians | NSCLC | MSP | Blood | 5 | 21 | 2 | 22 |  |
|  |  |  |  |  |  |  |  |  |  |  |  |
| *CDKN2B* |  |  |  |  |  |  |  |  |  |  |  |
|  | Jianlong Zhang 2011 | China | Asians | NSCLC | MSP | Tissue | 18 | 21 | 48 | 55 |  |
|  |  |  |  |  |  |  |  |  |  |  |  |
| *MIR34b/c* |  |  |  |  |  |  |  |  |  |  |  |
|  | Yushun Gao 2011 | China | Asians | NSCLC | MSP | Tissue | 34 | 97 | 27 | 53 |  |
|  |  |  |  |  |  |  |  |  |  |  |  |
| *ZO-1* |  |  |  |  |  |  |  |  |  |  |  |
|  | Yekai Wang (TI) 2011 | China | Asians | NSCLC | MSP | Tissue | 18 | 41 | 10 | 43 |  |
|  | Yekai Wang (BL) 2011 | China | Asians | NSCLC | MSP | Blood | 9 | 41 | 10 | 43 |  |
|  |  |  |  |  |  |  |  |  |  |  |  |
| *Caspase-8* |  |  |  |  |  |  |  |  |  |  |  |
|  | Qiang Zhang 2012 | China | Asians | NSCLC | MSP | Tissue | 19 | 24 | 19 | 45 |  |
|  |  |  |  |  |  |  |  |  |  |  |  |
| *HYAL1* |  |  |  |  |  |  |  |  |  |  |  |
|  | Dong Xie (TI) 2013 | China | Asians | NSCLC | MSP | Tissue | 17 | 36 | 18 | 35 |  |
|  | Dong Xie (BL) 2013 | China | Asians | NSCLC | MSP | Blood | 12 | 36 | 18 | 35 |  |
|  |  |  |  |  |  |  |  |  |  |  |  |
| *hOGG1* |  |  |  |  |  |  |  |  |  |  |  |
|  | Dong Xie (TI) 2013 | China | Asians | NSCLC | MSP | Tissue | 20 | 36 | 16 | 35 |  |
|  | Dong Xie (BL) 2013 | China | Asians | NSCLC | MSP | Blood | 15 | 36 | 15 | 35 |  |
|  |  |  |  |  |  |  |  |  |  |  |  |
| *CYP2J2* |  |  |  |  |  |  |  |  |  |  |  |
|  | Wanming Liao 2011 | China | Asians | NSCLC | BSP | Tissue | 5 | 15 | 20 | 35 |  |
|  |  |  |  |  |  |  |  |  |  |  |  |
| *Fas* |  |  |  |  |  |  |  |  |  |  |  |
|  | Yifei Zhu 2011 | China | Asians | NSCLC | MSP | Tissue | 12 | 21 | 14 | 23 |  |
|  |  |  |  |  |  |  |  |  |  |  |  |
| *IRF8*  *AKAP12*  *IGFBP4* | Makoto Suzuki 2014  Fengli Han 2014 | Japan  China | Asians  Asians | NSCLC  NSCLC | PSQ  MSP | Tissue  Tissue | 64  16 | 149    19 | 16  13 | 28  24 |  |
|  | Yong Li 2012 | China | Asians | NSCLC | MSP | Tissue | 24 | 41 | 23 | 51 |  |

AC stands for adenocarcinoma; SCC stands for squamous cell carcinoma; NSCLC stands for non-small cell lung cancer; M+ stands for methylated numbers. NS stands for not significant; S stands for significant; NA stands for not application;Total stands for the sum of methylated and unmethylated numbers from NSCLC patients. TI stands for tissue; BL stands for blood; SP stands for sputum; EBC stands for exhaled breath condensate sample; Frag_01 and Frag_02 stand for the differently methylated regions of EN1 gene. P16 and P14 stand for the isoform of the CDKN2A; M1,M5 and M9 stand for the differently methylated regions of IGSF4 ;m2a,m2b and m2a+m2b stand for the differently methylated regions of DAB2IP. The various methods of methylation analysis：PCR polymerase chain reaction; MSP methylation-specific polymerase chain reaction;QMSP quantitative methylation-specific polymerase chain reaction; COBRA combined bisulfite restriction; BSP Bisulfite Genomic Sequencing PCR; FRTPCR Fluorescence-based real-time PCR; PSQ Pyrosequencing; nMSP nested MSP; 3DPCR 3-Dimensional microarray based PCR; FMSP Fluorescent quantitative methylation-specific polymerase chain reaction; BS-MSP bisulfite conversion-specific methylation specific PCR; MS-HRM methylation-sensitive high resolution melting; RTMSP stands for real-time methylation-specific PCR; MPCR stands for multiplex PCR methylation assay; bisulfite SSCP stands for bisulfite single-strand conformational polymorphism.

**References**

1. Balgkouranidou I, Chimonidou M, Milaki G*, et al.* Breast cancer metastasis suppressor-1 promoter methylation in cell-free DNA provides prognostic information in non-small cell lung cancer. Br J Cancer 2014;110(8):2054-62.

2. Carvalho RH, Hou J, Haberle V*, et al.* Genomewide DNA methylation analysis identifies novel methylated genes in non-small-cell lung carcinomas. J Thorac Oncol 2013;8(5):562-73.

3. Chang YS, Wang L, Liu D*, et al.* Correlation between insulin-like growth factor-binding protein-3 promoter methylation and prognosis of patients with stage I non-small cell lung cancer. Clin Cancer Res 2002;8(12):3669-75.

4. Chen H, Suzuki M, Nakamura Y*, et al.* Aberrant methylation of RASGRF2 and RASSF1A in human non-small cell lung cancer. Oncol Rep 2006;15(5):1281-5.

5. Chen SC, Lin CY, Chen YH*, et al.* Aberrant promoter methylation of EDNRB in lung cancer in Taiwan. Oncol Rep 2006;15(1):167-72.

6. Chen Y, Cui T, Knosel T*, et al.* IGFBP7 is a p53 target gene inactivated in human lung cancer by DNA hypermethylation. Lung Cancer 2011;73(1):38-44.

7. Chun-hui Z. Detection and Analysis of Methylation Status of HIN-1 Genes in Non-small Cell Lung Cancer Tissues. The Journal of Practical Medicine 2012;28(2):266-268.

8. Chunyan K. Investigation on the relationgship between the promoter hypermethylation of MGMT and clinicopathologic feature in NSCLC tissues. Chinese Journal of Laboratory Diagnosis 2011;15(1):81-82.

9. Cong T. Correlation between RARβ gene promoter methylation and P53 gene mutations in non-small cell lung cancer. Chinese Journal of Medical Genetics 2012;29(2):131-136.

10. Conghui W. The methylation status of TMS1 gene in non-small cell lung cancer and its clinical significance. Acta Universitatis Medicinalis Anhui 2008;43(1):46-50.

11. Degan L. Significance of MGMT hypermethylation in serum DNA of non-small cell lung cancer patients. International Journal of Respiration 2009;29(20):1221-1224.

12. Degan L. Detection and significance of serum CDH13 gene methylation with non small cell lung cancer in patients. CHINA ONCOLOGY 2011;21(8):657-660.

13. De-gan L. Detection and significance of abnormal methylation of Ras association domain family 1A gene in serum of non-small cell lung cancer patients. International Journal of respiration 2010;30(18):1093-1096.

14. De-gan L. The relation between serum colorectal adenomatous polyposis genes methylayion and Non-small cell lung cancer. Journal of Chinese Physician 2010;12(1):82-84.

15. De-gan L. The detection and significance of serum death associated protein kinase gene aberrant methylation for the Non-small cell lung cancer patients. Shandong Medical Journal 2010;50(9):69-70.

16. De-gan L. Significance of RUNX3 Hypermethylation in Serum DNA of Non-small Cell Lung Cancer

Patients. Chinese Journal of Cancer Prevention and Treatment 2011;38(6):671-674.

17. De-gan L. Significance of TMSl／ASC methylation in serum of non-small cell lung cancer patients. International Journal of Respiration 2013;33(4):250-253.

18. Ekim M, Caner V, Buyukpinarbasili N*, et al.* Determination of O(6)-methylguanine DNA methyltransferase promoter methylation in non-small cell lung cancer. Genet Test Mol Biomarkers 2011;15(5):357-60.

19. Furonaka O, Takeshima Y, Awaya H*, et al.* Aberrant methylation and loss of expression of O-methylguanine-DNA methyltransferase in pulmonary squamous cell carcinoma and adenocarcinoma. Pathol Int 2005;55(6):303-9.

20. Geng X, Wang F, Zhang L*, et al.* Loss of heterozygosity combined with promoter hypermethylation, the main mechanism of human MutL Homolog (hMLH1) gene inactivation in non-small cell lung cancer in a Chinese population. Tumori 2009;95(4):488-94.

21. Gomes A, Reis-Silva M, Alarcao A*, et al.* Promoter hypermethylation of DNA repair genes MLH1 and MSH2 in adenocarcinomas and squamous cell carcinomas of the lung. Rev Port Pneumol 2014;20(1):20-30.

22. Gonzalez-Quevedo R, Garcia-Aranda C, Moran A*, et al.* Differential impact of p16 inactivation by promoter methylation in non-small cell lung and colorectal cancer: clinical implications. Int J Oncol 2004;24(2):349-55.

23. Gui-zhi L. Detection of RASSF1A hypermethylation in plasma a DNA from non-small cell lung cancer patients and its value in non-small cell lung cancer diagnosis. HENAN MEDICAL RESEARCH 2010;19(1):4-8.

24. Haizhu S. Analysis of SFRP1 promoter methylation status in non-small cell lung cancer patients. Chinese Clinical Oncology 2010;15(3):207-210.

25. Hanling F. Expression of SFRPl gene methylation and SFRPl protein in non-small celllung cancer. China Journal Surgonc 2012;4(2):95-98.

26. Heller G, Fong KM, Girard L*, et al.* Expression and methylation pattern of TSLC1 cascade genes in lung carcinomas. Oncogene 2006;25(6):959-68.

27. Heller G, Weinzierl M, Noll C*, et al.* Genome-wide miRNA expression profiling identifies miR-9-3 and miR-193a as targets for DNA methylation in non-small cell lung cancers. Clin Cancer Res 2012;18(6):1619-29.

28. Hong-li L. Effects of Methylation of FHIT Gene on it’s Protein and mRNA Expression

in Non-small Cell Lung Cancer. Chinese Journal Lung Cancer 2009;12(7):760-764.

29. Hsu HS, Wen CK, Tang YA*, et al.* Promoter hypermethylation is the predominant mechanism in hMLH1 and hMSH2 deregulation and is a poor prognostic factor in nonsmoking lung cancer. Clin Cancer Res 2005;11(15):5410-6.

30. Huafu Z. STUDY ON p16 GENE METHYLATION EXPRESSION IN NON-SMALL CELL LUNG CANCER. JOURNAL OF GUANGXI MEDICAL UNIVERSITY 2009;26(3):353-355.

31. Hui Z. Prognostic Value of Methylation Status of RASSF1A Gene as an Independent Factor of Non-small Cell Lung Cancer. China Journal of Lung Cancer 2010;13(4):311-316.

32. Hwang JA, Kim Y, Hong SH*, et al.* Epigenetic inactivation of heparan sulfate (glucosamine) 3-o-sulfotransferase 2 in lung cancer and its role in tumorigenesis. PLoS One 2013;8(11):e79634.

33. Jarmalaite S, Kannio A, Anttila S*, et al.* Aberrant p16 promoter methylation in smokers and former smokers with nonsmall cell lung cancer. Int J Cancer 2003;106(6):913-8.

34. Jianlong Z. Correlation between methylation of INK4/ARF locus and progression of non - small cell lung cancer. Guangdong Medical Journal 2011;32(19):2527-2530.

35. Jian-you H. Aberrent methylation of 5' CpG island in the p16 tumor suppressor gene

in non-small cell lung cancer. Journal of Guangdong Medical College 2001;19(5):321-322.

36. Jun W. Study on detection of aberrant promoter hypermethylation of p16 and DAP kinase in serum DNA from patients with non -small cell lung cancer. China Journal Lung Cancer 2002;5(3):188-190.

37. Kaira K, Sunaga N, Tomizawa Y*, et al.* Epigenetic inactivation of the RAS-effector gene RASSF2 in lung cancers. Int J Oncol 2007;31(1):169-73.

38. Kikuchi S, Yamada D, Fukami T*, et al.* Promoter methylation of DAL-1/4.1B predicts poor prognosis in non-small cell lung cancer. Clin Cancer Res 2005;11(8):2954-61.

39. Kim DH, Kim JS, Ji YI*, et al.* Hypermethylation of RASSF1A promoter is associated with the age at starting smoking and a poor prognosis in primary non-small cell lung cancer. Cancer Res 2003;63(13):3743-6.

40. Kim DS, Kim MJ, Lee JY*, et al.* Aberrant methylation of E-cadherin and H-cadherin genes in nonsmall cell lung cancer and its relation to clinicopathologic features. Cancer 2007;110(12):2785-92.

41. Kim DS, Kim MJ, Lee JY*, et al.* Epigenetic inactivation of Homeobox A5 gene in nonsmall cell lung cancer and its relationship with clinicopathological features. Mol Carcinog 2009;48(12):1109-15.

42. Kim DS, Lee JY, Lee SM*, et al.* Promoter methylation of the RGC32 gene in nonsmall cell lung cancer. Cancer 2011;117(3):590-6.

43. Kim DS, Lee SM, Yoon GS*, et al.* Infrequent hypermethylation of the PTEN gene in Korean non-small-cell lung cancers. Cancer Sci 2010;101(2):568-72.

44. Kim JS, Kim H, Shim YM*, et al.* Aberrant methylation of the FHIT gene in chronic smokers with early stage squamous cell carcinoma of the lung. Carcinogenesis 2004;25(11):2165-71.

45. Kobatake T, Yano M, Toyooka S*, et al.* Aberrant methylation of p57KIP2 gene in lung and breast cancers and malignant mesotheliomas. Oncol Rep 2004;12(5):1087-92.

46. Koga T, Takeshita M, Yano T*, et al.* CHFR hypermethylation and EGFR mutation are mutually exclusive and exhibit contrastive clinical backgrounds and outcomes in non-small cell lung cancer. Int J Cancer 2011;128(5):1009-17.

47. Kuroki T, Trapasso F, Yendamuri S*, et al.* Allelic loss on chromosome 3p21.3 and promoter hypermethylation of semaphorin 3B in non-small cell lung cancer. Cancer Res 2003;63(12):3352-5.

48. Lai JC, Cheng YW, Goan YG*, et al.* Promoter methylation of O(6)-methylguanine-DNA-methyltransferase in lung cancer is regulated by p53. DNA Repair (Amst) 2008;7(8):1352-63.

49. Lee SM, Choi JE, Na YK*, et al.* Genetic and epigenetic alterations of the LKB1 gene and their associations with mutations in TP53 and EGFR pathway genes in Korean non-small cell lung cancers. Lung Cancer 2013;81(2):194-9.

50. Lee SM, Lee JY, Choi JE*, et al.* Epigenetic inactivation of retinoid X receptor genes in non-small cell lung cancer and the relationship with clinicopathologic features. Cancer Genet Cytogenet 2010;197(1):39-45.

51. Lee SM, Park JY, Kim DS. Wif1 hypermethylation as unfavorable prognosis of non-small cell lung cancers with EGFR mutation. Mol Cells 2013;36(1):69-73.

52. Lei X. Expression of p16 genemethylation and protein in non- small cell lung cancer. Guangdong Medical Journal 2009;30(12):1816-1818.

53. Li L, Shen Y, Wang M*, et al.* Identification of the methylation of p14ARF promoter as a novel non-invasive biomarker for early detection of lung cancer. Clin Transl Oncol 2014;16(6):581-9.

54. Li N, Zhang F, Li S*, et al.* Epigenetic silencing of MicroRNA-503 regulates FANCA expression in non-small cell lung cancer cell. Biochem Biophys Res Commun 2014;444(4):611-6.

55. Li QL, Kim HR, Kim WJ*, et al.* Transcriptional silencing of the RUNX3 gene by CpG hypermethylation is associated with lung cancer. Biochem Biophys Res Commun 2004;314(1):223-8.

56. Li W, Deng J, Jiang P*, et al.* Association of 5'-CpG island hypermethylation of the FHIT gene with lung cancer in southern-central Chinese population. Cancer Biol Ther 2010;10(10):997-1000.

57. Liu RY, Lei Z, Li W*, et al.* Infrequently methylated event at sites -181 to -9 within the 5' CpG island of E-cadherin in non-small cell lung cancer. Exp Lung Res 2009;35(7):541-53.

58. Liu Y, Lan Q, Siegfried JM*, et al.* Aberrant promoter methylation of p16 and MGMT genes in lung tumors from smoking and never-smoking lung cancer patients. Neoplasia 2006;8(1):46-51.

59. Marsit CJ, Kim DH, Liu M*, et al.* Hypermethylation of RASSF1A and BLU tumor suppressor genes in non-small cell lung cancer: implications for tobacco smoking during adolescence. Int J Cancer 2005;114(2):219-23.

60. Marsit CJ, Liu M, Nelson HH*, et al.* Inactivation of the Fanconi anemia/BRCA pathway in lung and oral cancers: implications for treatment and survival. Oncogene 2004;23(4):1000-4.

61. Marsit CJ, Okpukpara C, Danaee H*, et al.* Epigenetic silencing of the PRSS3 putative tumor suppressor gene in non-small cell lung cancer. Mol Carcinog 2005;44(2):146-50.

62. Mengqing P. Detection of methylation and deletion of p16 gene in non-small cell lung cancer. China Journal Lung Cancer 2002;5(4):250-253.

63. Muscarella LA, Parrella P, D'Alessandro V*, et al.* Frequent epigenetics inactivation of KEAP1 gene in non-small cell lung cancer. Epigenetics 2011;6(6):710-9.

64. Na YK, Lee SM, Hong HS*, et al.* Hypermethylation of growth arrest DNA-damage-inducible gene 45 in non-small cell lung cancer and its relationship with clinicopathologic features. Mol Cells 2010;30(1):89-92.

65. Nan S. Methylation of p16 and RASSF1A Genes in Sputum Samples Associated with Peripheral Non-small Cell Lung Cancer. Progress in Modern Biomedicine 2012;12(13):2503-2510,2548.

66. Oleksiewicz U, Daskoulidou N, Liloglou T*, et al.* Neuroglobin and myoglobin in non-small cell lung cancer: expression, regulation and prognosis. Lung Cancer 2011;74(3):411-8.

67. Peng Z, Shan C, Wang H. [Value of promoter methylation of RASSF1A, p16, and DAPK genes in induced sputum in diagnosing lung cancers]. Zhong Nan Da Xue Xue Bao Yi Xue Ban 2010;35(3):247-53.

68. Powrozek T, Krawczyk P, Kucharczyk T*, et al.* Septin 9 promoter region methylation in free circulating DNA-potential role in noninvasive diagnosis of lung cancer: preliminary report. Med Oncol 2014;31(4):917.

69. Qing L. The combined detection of serum DAPK in patients with non small cell lung cancer gene, p16 gene methylation. Chinese Clinical of Geriatrics 2006;26(10):1304-1306.

70. Qing X. Diagnostic value of methylation of p16 genes in patients with non-small cell lung cancer. Clinical Medicine of China 2008;24(6):521-523.

71. Saito K, Kawakami K, Matsumoto I*, et al.* Long interspersed nuclear element 1 hypomethylation is a marker of poor prognosis in stage IA non-small cell lung cancer. Clin Cancer Res 2010;16(8):2418-26.

72. Sato K, Tomizawa Y, Iijima H*, et al.* Epigenetic inactivation of the RUNX3 gene in lung cancer. Oncol Rep 2006;15(1):129-35.

73. Schmiemann V, Bocking A, Kazimirek M*, et al.* Methylation assay for the diagnosis of lung cancer on bronchial aspirates: a cohort study. Clin Cancer Res 2005;11(21):7728-34.

74. Seng TJ, Currey N, Cooper WA*, et al.* DLEC1 and MLH1 promoter methylation are associated with poor prognosis in non-small cell lung carcinoma. Br J Cancer 2008;99(2):375-82.

75. Shigematsu H, Suzuki M, Takahashi T*, et al.* Aberrant methylation of HIN-1 (high in normal-1) is a frequent event in many human malignancies. Int J Cancer 2005;113(4):600-4.

76. Shimamoto T, Ohyashiki JH, Hirano T*, et al.* Hypermethylation of E-cadherin gene is frequent and independent of p16INK4A methylation in non-small cell lung cancer: potential prognostic implication. Oncol Rep 2004;12(2):389-95.

77. Shivapurkar N, Toyooka S, Toyooka KO*, et al.* Aberrant methylation of trail decoy receptor genes is frequent in multiple tumor types. Int J Cancer 2004;109(5):786-92.

78. SONG H. DNA Methylation of Tumour Suppressor Genes Located on Chromosome 3p in Non-small Cell Lung Cancer. Chinese Journal of Lung Cancer 2011;14(3):233-238.

79. Suga Y, Miyajima K, Oikawa T*, et al.* Quantitative p16 and ESR1 methylation in the peripheral blood of patients with non-small cell lung cancer. Oncol Rep 2008;20(5):1137-42.

80. Suzuki M, Iizasa T, Nakajima T*, et al.* Aberrant methylation of IL-12Rbeta2 gene in lung adenocarcinoma cells is associated with unfavorable prognosis. Ann Surg Oncol 2007;14(9):2636-42.

81. Suzuki M, Mohamed S, Nakajima T*, et al.* Aberrant methylation of CXCL12 in non-small cell lung cancer is associated with an unfavorable prognosis. Int J Oncol 2008;33(1):113-9.

82. Suzuki M, Shigematsu H, Nakajima T*, et al.* Synchronous alterations of Wnt and epidermal growth factor receptor signaling pathways through aberrant methylation and mutation in non small cell lung cancer. Clin Cancer Res 2007;13(20):6087-92.

83. Suzuki M, Shigematsu H, Shames DS*, et al.* Methylation and gene silencing of the Ras-related GTPase gene in lung and breast cancers. Ann Surg Oncol 2007;14(4):1397-404.

84. Suzuki M, Shiraishi K, Eguchi A*, et al.* Aberrant methylation of LINE-1, SLIT2, MAL and IGFBP7 in non-small cell lung cancer. Oncol Rep 2013;29(4):1308-14.

85. Takeshita M, Koga T, Takayama K*, et al.* Alternative efficacy-predicting markers for paclitaxel instead of CHFR in non-small-cell lung cancer. Cancer Biol Ther 2010;10(9):933-41.

86. Tan S, Sun C, Wei X*, et al.* Quantitative assessment of lung cancer associated with genes methylation in the peripheral blood. Exp Lung Res 2013;39(4-5):182-90.

87. Tang Y, Wu F, Hu C. [RUNX3 promoter hypermethylation and prognosis of early surgically resected non-small cell lung cancers]. Zhong Nan Da Xue Xue Bao Yi Xue Ban 2011;36(7):650-4.

88. Tian L, Suzuki M, Nakajima T*, et al.* Clinical significance of aberrant methylation of prostaglandin E receptor 2 (PTGER2) in nonsmall cell lung cancer: association with prognosis, PTGER2 expression, and epidermal growth factor receptor mutation. Cancer 2008;113(6):1396-403.

89. Topaloglu O, Hoque MO, Tokumaru Y*, et al.* Detection of promoter hypermethylation of multiple genes in the tumor and bronchoalveolar lavage of patients with lung cancer. Clin Cancer Res 2004;10(7):2284-8.

90. Ulivi P, Zoli W, Calistri D*, et al.* p16INK4A and CDH13 hypermethylation in tumor and serum of non-small cell lung cancer patients. J Cell Physiol 2006;206(3):611-5.

91. van den Berg RM, Snijders PJ, Grunberg K*, et al.* Comprehensive CADM1 promoter methylation analysis in NSCLC and normal lung specimens. Lung Cancer 2011;72(3):316-21.

92. Wang J, Lee JJ, Wang L*, et al.* Value of p16INK4a and RASSF1A promoter hypermethylation in prognosis of patients with resectable non-small cell lung cancer. Clin Cancer Res 2004;10(18 Pt 1):6119-25.

93. Wang R, Zhang YW, Chen LB. Aberrant promoter methylation of FBLN-3 gene and clinicopathological significance in non-small cell lung carcinoma. Lung Cancer 2010;69(2):239-44.

94. Wang Y, Zhang D, Zheng W*, et al.* Multiple gene methylation of nonsmall cell lung cancers evaluated with 3-dimensional microarray. Cancer 2008;112(6):1325-36.

95. Wang YC, Lu YP, Tseng RC*, et al.* Inactivation of hMLH1 and hMSH2 by promoter methylation in primary non-small cell lung tumors and matched sputum samples. J Clin Invest 2003;111(6):887-95.

96. Wanmin L. Promoter hypermethylation status of CDH13 gene in lung cancer. Modern Medicine Journal of China 2011;13(3):8-10.

97. Wei WL, Hu HY, Zhang LJ*, et al.* [Promoter methylation of ASPP1 and ASPP2 genes in non-small cell lung cancers]. Zhonghua Bing Li Xue Za Zhi 2011;40(8):532-6.

98. Wen Z. The study of P16 methylation and P16 Protein expression in non-small cell lung cancers[NSCLC] Journal of General Hospital of Air Force 2004;20(1):24-30.

99. Wenhu T. The effects of p16 genemethylation on the risk of non- small cell lung cancer. ONCOLOGY PROGRESS 2007;5(4):393-403.

100. Wu D, Xiong L, Wu S*, et al.* TFPI-2 methylation predicts poor prognosis in non-small cell lung cancer. Lung Cancer 2012;76(1):106-11.

101. Wu JY, Wang J, Lai JC*, et al.* Association of O6-methylguanine-DNA methyltransferase (MGMT) promoter methylation with p53 mutation occurrence in non-small cell lung cancer with different histology, gender, and smoking status. Ann Surg Oncol 2008;15(11):3272-7.

102. Wu YH, Tsai Chang JH, Cheng YW*, et al.* Xeroderma pigmentosum group C gene expression is predominantly regulated by promoter hypermethylation and contributes to p53 mutation in lung cancers. Oncogene 2007;26(33):4761-73.

103. Xiao P, Chen JR, Zhou F*, et al.* Methylation of P16 in exhaled breath condensate for diagnosis of non-small cell lung cancer. Lung Cancer 2014;83(1):56-60.

104. Xiao-fang S. The significance of methylation in lung cancer. Chinese Journal of Laboratory Diagnosis 2012;16(5):836-839.

105. Xinarianos G, McRonald FE, Risk JM*, et al.* Frequent genetic and epigenetic abnormalities contribute to the deregulation of cytoglobin in non-small cell lung cancer. Hum Mol Genet 2006;15(13):2038-44.

106. Yanagawa N, Tamura G, Oizumi H*, et al.* Promoter hypermethylation of RASSF1A and RUNX3 genes as an independent prognostic prediction marker in surgically resected non-small cell lung cancers. Lung Cancer 2007;58(1):131-8.

107. Yang J, Shen Y, Liu B*, et al.* Promoter methylation of BRMS1 correlates with smoking history and poor survival in non-small cell lung cancer patients. Lung Cancer 2011;74(2):305-9.

108. Yang LH, Xu HT, Li QC*, et al.* Abnormal hypermethylation and clinicopathological significance of Axin gene in lung cancer. Tumour Biol 2013;34(2):749-57.

109. Yang TM, Leu SW, Li JM*, et al.* WIF-1 promoter region hypermethylation as an adjuvant diagnostic marker for non-small cell lung cancer-related malignant pleural effusions. J Cancer Res Clin Oncol 2009;135(7):919-24.

110. Yano M, Toyooka S, Tsukuda K*, et al.* Aberrant promoter methylation of human DAB2 interactive protein (hDAB2IP) gene in lung cancers. Int J Cancer 2005;113(1):59-66.

111. Yanrong L. The serum DKK3 gene promoter methylation is associated with the Non-small cell lung cancer. International Journal of Laboratory Medicine 2013;34(11):1450-1452.

112. Yekai W. Analysis and clinical significance of methylation status of zonula occluden-1

promoter in patients with non-small cell lung cancer. ACTA ANATOMICA SINICA 2011;42(2):195-200.

113. Ying L. PromoterHypermethylation ofMGMT Gene and Expression

ofK- ras in Human Non- small- cell Lung Cancer. Journal of Medical Forum 2010;31(13):1-4.

114. Yong J. Value of RUNX3 gene promoter methylation in diagnosis of non-small cellular lung cancer Jangsu Medical Jounal 2012;38(4):419-422.

115. Yong L. Identification of Hyper-Methylated Sequence in Lung Cancer by Methylated CpG Island Amplification Coupled with Representationa Difference Analysis. Chinese General Practice 2012;15(2C):644-648.

116. Yong-qiang D. Relationship between non-small cell lung cancer and the aberrant methylation of cytosine-phosphoric

acid-guanine motif island of tissue factor pathway inhibitor 2 gene. Chinese Journal of Experimental Surgery 2013;30(5):986-988.

117. Yoshino M, Suzuki M, Tian L*, et al.* Promoter hypermethylation of the p16 and Wif-1 genes as an independent prognostic marker in stage IA non-small cell lung cancers. Int J Oncol 2009;35(5):1201-9.

118. Yu ZH, Wang YC, Chen LB*, et al.* [Analysis of RASSF1A promoter hypermethylation in serum DNA of non-small cell lung cancer]. Zhonghua Zhong Liu Za Zhi 2008;30(4):284-7.

119. Yunming K. Detection and significance of promoter hypermethylation of p16 gene and MGMT gene in plasma from non-small cell lung cancer patients. Tumor 2007;27(9):715-718.

120. Zhang Y, Miao Y, Yi J*, et al.* Frequent epigenetic inactivation of deleted in lung and esophageal cancer 1 gene by promoter methylation in non-small-cell lung cancer. Clin Lung Cancer 2010;11(4):264-70.

121. Zhang YW, Miao YF, Yi J*, et al.* Transcriptional inactivation of secreted frizzled-related protein 1 by promoter hypermethylation as a potential biomarker for non-small cell lung cancer. Neoplasma 2010;57(3):228-33.

122. Zheng-hong Y. Analysis of Methylation Status of RASSF1A Promoter Region and Transcription Level of Downstream Genes in non-Small Cell Lung Cancer. Journal of Modern Laboratory Medicine 2007;22(1):15-17.

123. Zhenhua Y. Promoter hyperme thylat ion of 11 tumor-related genes in non-small cell lung cancers. Journal of Clinical Internal Medicine 2005;22(10):708-710.

124. Zhen-hua Y. Methylation of antioncogen at 3p in non-small cell lung cancer. Chinese Journal of Cancer Prevention and Treatment 2007;14(5):363-365.

125. Zhen-hua Y. Methylation of Promoter for hMLH1 in Non-small Cell Lung Cancer. Chinese Journal of Cancer Prevention and Treatment 2007;34(1):11-13.

126. Zhenxue B. The correlation between promoter region methylation as well as mRNA expression of PTEN gene and invasion as well as metastasis of non-small cell lung cancer. Hebei Medical Journal 2013;35(10):1452-1454.

127. Zhi-liang H. The methylation status detected and analysis in clinic about p14ARF

from non-small lung cancer tissues and its surrounding normal part. Journal of Jining Medical College 2014;37(1):27-36.

128. Dong X. Research for Epigenetic Mechanism of Lung Cancer Clinical research. The Second Military Medical University Department of Cardiothoracic Surgery ,The Second Military Medical University,Shanghai, 200003, China 2013.

129. Huafu Z. STUDY ON p16 GENE METHYLATION EXPRESSION IN NON-SMALL CELL LUNG CANCER clinical research. Guangxi Medical University Cardiothoracic surgery,Guangxi Medical University,Guangxi,530021,China 2007.

130. Kai-hua T. Alterations of The p14_ARF Gene in Non-Small Cell Lung Cancer and The Correlation of Its Expression With p16_INE4a,p53 Protein Clinical research. UNIVERSITY OF SCIENCE AND TECHNOLOGY OF CHINA Department of Cardiothoracic Surgey, The Affiliated Hospital of Medical College Qingdao University, Qingdao,266021, China 2004.

131. Liang Z. Study on the effect of hMLH1, p16, FHIT gene in the pathogenesis of non small cell lung cancer clinical research. Medical University Of Tianjin Oncology,Medical University Of Tianjin,Tianjin,300070,China 2003.

132. Lijian Z. Preliminary discussion on radiotherapy target guidance in non small cell lung cancer detection of P16 gene methylation clinical research. Qiingdao University Oncology,Qiingdao University,Qingdao,Shandong,266071 ,China 2007.

133. Ming L. Aberrant Methylation of the p16 and MGMT Genes Promoter

Region and Its Diagnostic Value in Lung Cancer Clinical research. Hebei Medical University Department of Surgery, Hebei Medical University, Shijiazhuang,Hebei,050017, China 2004.

134. Peng C. Non-samll cell lung cancer with plasma DAPK gene methylation relations Clinical research. Qingdao University Qingdao University Medical College , Qingdao , Shandong , 266071, China 2012.

135. Qiang Z. Studyon the Correlation between Methylation of

Caspase-8 Gene and Expression of FasL、Caspase-8

orol" in Non-small cell lung cancerorotein cell Cancer Clinical research. Suzhou University Department of Respiratory, Suzhou University, Suzhou，Jiangsu,215006, China 2012.

136. Qi-xin Z. The Methylation Status of E-cadherin Gene in

Non-small Cell Lung Cancer and Its Clinical

Significance Clinical research. Suzhou University Department of Respiratory,Suzhou University, Suzhou,Jiangsu,215006, China 2012.

137. Qunfeng Y. The Method of Quantitative Analysis DNA Hypermethylation and Its Application clinical research. Huazhong University of Science and Technology Labour health and sanitation,Huazhong University of Science and Technology,Wuhan,Hubei,430074,China 2004.

138. Sunyu G. Phase I study of non small cell lung cancer miRNA-34b/c encoding DNA methylation clinical research. Beijing Union Medical College Oncology,Beijing Union Medical College,Beijing,100730,China 2011.

139. Wanmin L. Aberrant promoter hypermethylation of CYP2J2 gene in lung cancer Clinical research. HUAZHONG UNIVERSITY OF SCIENCE AND TECHNOLOGY Department of Cardiology,Tongji Hospital Affiliated to Tongji Medical College of Huazhong University of Science and Technology,Wuhan,Hubei,430030,China 2011.

140. Wen-wen L. Study on Methylated Mechanisms Underlying mRNA

Expression of Several Tumor Suppressor Genes and Roles of

Demethylation in Non—Small Cell Lung Cancer Clinical research. Suzhou University Department of Respiratory, Suzhou University, Suzhou，Jiangsu，215006, China 2008.

141. Xiaochen W. Experimental study on the relationship between promoter region methylation status and polymorphism and non small cell lung cancer EPHB4 gene promoter clinical research. Soochow University Cardiothoracic surgery,Soochow University,Suzhou,Jiangsu,215000,China 2009.

142. Yanhua L. Study of the status of promoter methylation of DLEC1 and the expression of DLEC1 protein and Ki-67 antigen in non-small-cell lung cancer clinical research. Nanchang University Nanchang University,Nanchang,Jiangxi,330031,China 2010.

143. Yi-fei Z. Analysis of Correlation between Methylation of Fas Gene and Expression of Fas protein and Fas mRNA in tissues of Lung Cancer Clinical Research. SOOCHOW UNIVERSITY School of life science , Soochow University , Suzhou , 215000, China 2011.

144. Ying L. The promoter hypermethylation rate of MGMT in non-small-cell lung cancer and relationship with K-ras Master. Zhengzhou University Department of Respiratory, The First Affiliated Hospital of Zhengzhou University, Zhengzhou,Henan,450052, China 2006.

145. Ying L. Expression and Methylation of TFPI-2 in human non-small-cell lung cancinoma and the correlation with invasion and metastasis of neoplasms clinical research. Nanchang University Department of Respiratory,Nanchang University,Nanchang,Jiangxi,330031,China 2012.

146. Zhenhua Y. Study on the correlation between the 28 tumor related gene promoter CpG island methylation and transcription and non small cell lung cancer clinical research. Fudan University respiratory department,Fudan University,Shanghai,200433,China 2004.

147. Zhi-ming S. Significance of p16 Gene and MGMT hypermethylation in serum DNA of non-small cell lung cancer patients Clinical Research. NANCHANG UNIVERSITY Department of Fiberbronchoscopy,The First Affiliated Hospital Of Nanchang University,Nanchang,Jiangxi,330006 ,China 2011.

148. Zhongqi L. The Significance of Promoter Hypermethylation of p16 Gene in Circulating DNA on Early Diagnosis of Non-small Cell Lung Cancer Clinical research. BINZHOU MEDICAL UNIVERSITY Department of Respiratory,Yantai YuHuangDing Hospital,Yantai,Shandong,264000 ,China 2010.

149. Suzuki M, Ikeda K, Shiraishi K, et al. Aberrant methylation and silencing of expression in non-small cell lung cancer. Oncol Lett. 2014;8: 1025-1030.

150. Drilon A, Sugita H, Sima CS, et al. A prospective study of tumor suppressor gene methylation as a prognostic biomarker in surgically resected stage I to IIIA non-small-cell lung cancers. J Thorac Oncol. 2014;9: 1272-1277.

151. Fengli Han, Tao Xin, Liqiang Song, et al. Expression and methylation of AKAP12 gene in non small cell lung cancer. Modern Oncology 2014;22(08):1815-1818.

152. Harada H, Miyamoto K, Yamashita Y, Taniyama K, Mihara K, et al. (2015) Prognostic signature of protocadherin 10 methylation in curatively resected pathological stage I non-small-cell lung cancer. Cancer Med 4: 1536-1546.

153. Pan ZY, Jiang ZS, Ouyang HQ (2015) Study of the methylation patterns of the EGFR gene promoter in non-small cell lung cancer. Genet Mol Res 14: 9813-9820.

154. Zhang X, Yang X, Wang J, Liang T, Gu Y, et al. (2015) Down-regulation of PAX6 by promoter methylation is associated with poor prognosis in non small cell lung cancer. Int J Clin Exp Pathol 8: 11452-11457.
