# Supplementary material for: Distinguishing Lung Adenocarcinoma from Lung Squamous Cell Carcinoma by Two Hypomethylated and Three Hypermethylated Genes: A Meta-Analysis
Source: PLoS One. 2016 Feb 10;11(2):e0149088. doi: 10.1371/journal.pone.0149088 (PMC4749211; doi:10.1371/journal.pone.0149088)
Supplement: S2 Table — (DOC) [file pone.0149088.s003.doc]

S2 Table: Genes with less than 3 methylation studies.

| Gene | Studies | Overall OR (95% CI) | I^2^ | P value |
| --- | --- | --- | --- | --- |
| *SFRP5* | 2 | 0.92 [0.54, 1.56] | 0% | 0.75 |
| *SFRP2* | 2 | 2.74 [1.63, 4.60] | 0% | 0.0001 |
| *LINE-1* | 2 | 1.72 [0.25, 11.90] | 82% | 0.58 |
| *IL-12RB2* | 2 | 0.55 [0.32, 0.92] | 0% | 0.02 |
| *TIMP3* | 2 | 2.55 [0.27, 23.82] | 62% | 0.41 |
| *IGSF4* | 2 | 0.69 [0.44, 1.09] | 49% | 0.11 |
| *RARB2* | 2 | 0.47 [0.15, 1.43] | 0% | 0.18 |
| *PTGER2* | 2 | 1.91 [1.14, 3.21] | 0% | 0.01 |
| *BRCA1* | 2 | 3.27 [0.55, 19.43] | 24% | 0.19 |
| *MSH2* | 2 | 1.44 [0.69, 3.00] | 0% | 0.33 |
| *CXCL12* | 2 | 0.78 [0.46, 1.30] | 0% | 0.34 |
| *CHFR* | 2 | 0.14 [0.03, 0.61] | 63% | 0.009 |
| *BLU* | 2 | 2.13 [1.34, 3.38] | 0% | 0.001 |
| *EPB41L3* | 2 | 0.68 [0.25, 1.89] | 71% | 0.46 |
| *BRMS1* | 2 | 1.03 [0.45, 2.34] | 58% | 0.94 |
| *SEPT9* | 2 | 1.33 [0.49, 3.62] | 42% | 0.58 |
| *PTEN* | 2 | 0.82 [0.40, 1.69] | 0% | 0.6 |
| *Dkk3* | 2 | 1.57 [0.82, 3.01] | 32% | 0.18 |
| *HIN-1* | 2 | 0.80 [0.52, 1.25] | 0% | 0.33 |
| *TMS1* | 2 | 1.66 [0.69, 4.00] | 0% | 0.26 |
| *GSTP1* | 1 | 2.84 [0.13, 61.89] | NA | 0.51 |
| *IGFBP3* | 1 | 1.67 [0.65, 4.30] | NA | 0.29 |
| *CDKN2B* | 1 | 1.55 [0.06, 42.91] | NA | 0.8 |
| *HPP1* | 1 | 0.97 [0.20, 4.59] | NA | 0.97 |
| *DRM/Gremlin* | 1 | 0.38 [0.07, 2.11] | NA | 0.27 |
| *Reprimo* | 1 | 0.29 [0.07, 1.21] | NA | 0.09 |
| *SPARC* | 1 | 0.97 [0.20, 4.59] | NA | 0.97 |
| *EDNRB* | 1 | 0.35 [0.13, 0.98] | NA | 0.05 |
| *KEAP1* | 1 | 0.41 [0.06, 3.01] | NA | 0.38 |
| *EFEMP1* | 1 | 1.49 [0.45, 4.89] | NA | 0.52 |
| *SLIT2* | 1 | 2.67 [0.63, 11.38] | NA | 0.19 |
| *CYGB* | 1 | 3.00 [0.97, 9.30] | NA | 0.06 |
| *ESR1* | 1 | 1.23 [0.48, 3.19] | NA | 0.67 |
| *SEMA3B* | 1 | 1.75 [0.31, 10.02] | NA | 0.53 |
| *MIR9-3* | 1 | 4.62 [1.87, 11.42] | NA | 0.0009 |
| *RRAD* | 1 | 0.61 [0.34, 1.11] | NA | 0.1 |
| *RASGRF2* | 1 | 0.71 [0.29, 1.74] | NA | 0.46 |
| *TNFRSF10C* | 1 | 1.70 [0.16, 18.02] | NA | 0.66 |
| *FANCF* | 1 | 0.62 [0.23, 1.68] | NA | 0.35 |
| *DAB2IP* | 1 | 0.53 [0.29, 0.98] | NA | 0.04 |
| *RXRG* | 1 | 0.89 [0.40, 1.94] | NA | 0.76 |
| *RGC32* | 1 | 1.68 [0.78, 3.62] | NA | 0.19 |
| *GADD45A* | 1 | 3.90 [0.18, 82.82] | NA | 0.38 |
| *GADD45B* | 1 | 0.30 [0.07, 1.21] | NA | 0.09 |
| *GADD45G* | 1 | 1.00 [0.49, 2.06] | NA | 1 |
| *HOXA5* | 1 | 1.40 [0.60, 3.30] | NA | 0.44 |
| *NGB* | 1 | 5.05 [2.46, 10.34] | NA | < 0.00001 |
| *sFRP* | 1 | 1.58 [0.89, 2.79] | NA | 0.12 |
| *RXRA* | 1 | 1.28 [0.29, 5.60] | NA | 0.74 |
| *RXRB* | 1 | 0.36 [0.06, 2.06] | NA | 0.25 |
| *MIR-193a* | 1 | 0.58 [0.26, 1.33] | NA | 0.2 |
| *Axin* | 1 | 0.68 [0.26, 1.79] | NA | 0.43 |
| *MAL* | 1 | 0.65 [0.15, 2.71] | NA | 0.55 |
| *LKB1* | 1 | 1.33 [0.49, 3.66] | NA | 0.58 |
| *MIR-503* | 1 | 0.73 [0.22, 2.49] | NA | 0.62 |
| *CD44* | 1 | 0.92 [0.07, 12.28] | NA | 0.95 |
| *TERT* | 1 | 0.67 [0.11, 4.21] | NA | 0.67 |
| *CALCA* | 1 | 0.46 [0.04, 5.09] | NA | 0.53 |
| *ER* | 1 | 2.86 [0.42, 19.65] | NA | 0.29 |
| *TNFRSF10D* | 1 | 0.67 [0.13, 3.52] | NA | 0.63 |
| *ASPP1* | 1 | 1.35 [0.55, 3.34] | NA | 0.51 |
| *p57KIP2* | 1 | 1.04 [0.36, 3.01] | NA | 0.94 |
| *PRSS3* | 1 | 0.89 [0.46, 1.74] | NA | 0.73 |
| *XPC* | 1 | 0.95 [0.49, 1.83] | NA | 0.87 |
| *RASSF2* | 1 | 1.41 [0.58, 3.40] | NA | 0.45 |
| *HS3ST2* | 1 | 1.71 [1.02, 2.86] | NA | 0.04 |
| *ZIC4* | 1 | 0.22 [0.05, 0.95] | NA | 0.04 |
| *DOT1L* | 1 | 0.06 [0.00, 1.24] | NA | 0.07 |
| *LIMK1* | 1 | 0.24 [0.06, 0.86] | NA | 0.03 |
| *EN1* | 1 | 0.27 [0.10, 0.76] | NA | 0.01 |
| *MSC* | 1 | 9.04 [2.18, 37.50] | NA | 0.002 |
| *GAS1* | 1 | 7.07 [1.34, 37.22] | NA | 0.02 |
| *HOXA1* | 1 | 5.33 [1.18, 24.18] | NA | 0.03 |
| *FAM78B* | 1 | 10.27 [2.63, 40.12] | NA | 0.0008 |
| *MYOD1* | 1 | 1.95 [0.56, 6.73] | NA | 0.29 |
| *WT1* | 1 | 0.76 [0.23, 2.51] | NA | 0.65 |
| *TP73* | 1 | 2.46 [0.77, 7.79] | NA | 0.13 |
| *AR* | 1 | 3.17 [0.69, 14.46] | NA | 0.14 |
| *p21WAF* | 1 | 0.41 [0.10, 1.66] | NA | 0.21 |
| *0CT6* | 1 | 1.06 [0.26, 4.37] | NA | 0.93 |
| *EPHB4* | 1 | 1.79 [0.71, 4.47] | NA | 0.21 |
| *CDKN2B* | 1 | 0.88 [0.20, 3.76] | NA | 0.86 |
| *MIR34b/c* | 1 | 0.52 [0.26, 1.03] | NA | 0.06 |
| *ZO-1* | 1 | 1.58 [0.58, 4.31] | NA | 0.37 |
| *Caspase-8* | 1 | 5.20 [1.65, 16.41] | NA | 0.005 |
| *HYAL1* | 1 | 0.64 [0.33, 1.24] | NA | 0.18 |
| *PAX6* | 1 | 1.79 [0.71, 4.47] | NA | 0.21 |
| *PCDH10* | 1 | 0.76 [0.23, 2.51] | NA | 0.65 |
| *EGFR* | 1 | 0.27 [0.10, 0.76] | NA | 0.01 |
| *hOGG1* | 1 | 1.19 [0.61, 2.31] | NA | 0.6 |
| *CYP2J2* | 1 | 0.38 [0.11, 1.33] | NA | 0.13 |
| *Fas* | 1 | 0.86 [0.26, 2.86] | NA | 0.8 |
| *IGFBP4* | 1 | 1.72 [0.75, 3.94] | NA | 0.2 |
| *IRF8* | 1 | 0.56 [0.25, 1.28] | NA | 0.3 |
| *AKAP12* | 1 | 4.51 [1.04, 19.66] | NA | 0.35 |
